# Whole-Exome Sequencing Reveals a Rapid Change in the Frequency of Rare Functional Variants in a Founding Population of Humans

**DOI:** 10.1371/journal.pgen.1003815

**Published:** 2013-09-26

**Authors:** Ferran Casals, Alan Hodgkinson, Julie Hussin, Youssef Idaghdour, Vanessa Bruat, Thibault de Maillard, Jean-Cristophe Grenier, Elias Gbeha, Fadi F. Hamdan, Simon Girard, Jean-François Spinella, Mathieu Larivière, Virginie Saillour, Jasmine Healy, Isabel Fernández, Daniel Sinnett, Jacques L. Michaud, Guy A. Rouleau, Elie Haddad, Françoise Le Deist, Philip Awadalla

**Affiliations:** 1 Centre de Recherche du Centre Hospitalier Universitaire Sainte-Justine, Université de Montréal, Montréal, Québec, Canada; 2 Centre d'Excellence en Neuromique de l'Université de Montréal, Centre de Recherche du Centre Hospitalier de l'Université de Montréal, Montréal, Québec, Canada; 3 Département de Microbiologie et Immunologie, Faculté de Médecine, Université de Montréal, Montréal, Québec, Canada; 4 Département de Pédiatrie, Faculté de Médecine, Université de Montréal, Montréal, Québec, Canada; 5 Département de Médecine, Faculté de Médecine, Université de Montréal, Montréal, Québec, Canada; 6 Montreal Neurological Institute and Hospital, McGill University, Montréal, Québec, Canada; 7 Department of Biochemistry, McGill University, Montréal, Québec, Canada; Dartmouth College, United States of America

## Abstract

Whole-exome or gene targeted resequencing in hundreds to thousands of individuals has shown that the majority of genetic variants are at low frequency in human populations. Rare variants are enriched for functional mutations and are expected to explain an important fraction of the genetic etiology of human disease, therefore having a potential medical interest. In this work, we analyze the whole-exome sequences of French-Canadian individuals, a founder population with a unique demographic history that includes an original population bottleneck less than 20 generations ago, followed by a demographic explosion, and the whole exomes of French individuals sampled from France. We show that in less than 20 generations of genetic isolation from the French population, the genetic pool of French-Canadians shows reduced levels of diversity, higher homozygosity, and an excess of rare variants with low variant sharing with Europeans. Furthermore, the French-Canadian population contains a larger proportion of putatively damaging functional variants, which could partially explain the increased incidence of genetic disease in the province. Our results highlight the impact of population demography on genetic fitness and the contribution of rare variants to the human genetic variation landscape, emphasizing the need for deep cataloguing of genetic variants by resequencing worldwide human populations in order to truly assess disease risk.

## Author Summary

Recent resequencing of the whole genome or the coding part of the genome (the exome) in thousands of individuals has described a large excess of low frequency variants in humans, probably arising as a consequence of recent rapid growth in human population sizes. Most rare variants are private to specific populations and are enriched for functional mutations, thus potentially having some medical relevance. In this study, we analyze whole-exome sequences from over a hundred individuals from the French-Canadian population, which was founded less than 400 years ago by about 8,500 French settlers who colonized the province between the 17th and 18th centuries. We show that in a remarkably short period of time this population has accumulated substantial differences, including an excess of rare, functional and potentially damaging variants, when compared to the original European population. Our results show the effects of population history on genetic variation that may have an impact on genetic fitness and disease, and have implications in the design of genetic studies, highlighting the importance of extending deep resequencing to worldwide human populations.

## Introduction

Genetic variation in humans is a result of stochastic processes, selection and demographic history [1]. Modern humans show a reduced level of differentiation due to recent population dispersion less than 100,000 years ago, and differences between populations are thought to account for little more than 15% of all genetic variation across individuals [2]. However, this picture is based on the allele frequency differences of common and shared variants between populations, representing only a small fraction of the total number of variants. Recently, much effort has been put into the description of the total variation landscape in human populations by resequencing hundreds to thousands of individuals from the same population at particular loci or for complete exomes [3]–[8]. Additionally, the 1000 Genomes Project has characterized the complete genomic sequences of more than one thousand humans covering worldwide diversity [9], [10].

Two important conclusions have arisen from studies deeply characterizing the allele frequency spectrum in human populations. First, the high number of low frequency variants is likely only explainable by models of recent demographic explosion [3]–[8]. Furthermore, low frequency variants are enriched for functional variants, particularly for nucleotide changes that affect protein function, and are therefore putatively more related to disease [3]–[8], [11]. Second, most rare variants are private or show very little sharing among continents [7], [8], [12], [13]. This may be particularly important in terms of genetic fitness, since rare variants are enriched for deleterious alleles. However, until now differences in the relative amount of detrimental variants have only been shown over relatively large timescales by comparing African and European populations [14], [15]. Furthermore, these findings predict a lack of replication in association studies using rare functional variants across populations, since rare variants can show higher levels of stratification [16], thus emphasizing the need of population-specific catalogues of genetic variation [12].

In this work, we analyze whole-exome sequence data from French-Canadian individuals, comparing various population level statistics to those for French and European populations, which allow us to make inferences about the fitness of a population with a unique demographic history. The current population of six million French-Canadians in Quebec are descendants of about 8,500 French settlers who colonized the province between 1608 and 1759, before the English conquest [17], [18]. Although colonization included emigrants from all of France, the migration event mostly originated from the Atlantic coast and Paris region. After 1760, French immigration virtually stopped, and the French-Canadian population experienced rapid growth due to a high birth rate, and became genetically isolated from France with limited exchange with other non-French communities in the same geographical area [19]. Overall, French-Canadians have experienced a growth from 8,500 to six million individuals, which represents a population expansion of more 700% in less than 20 generations. While other colonized territories in America or Oceania may have experienced a similar growth, the uniqueness of the French-Canadian population is due in part to the reduced contribution of new immigration after the first settlers [20] and the founding population is estimated to have contributed 90% of the current French-Canadian genetic pool [21]. In addition, during the 19th century new territories were colonized by a reduced number of settlers, contributing massively to the genetic pool in these regions, giving place to several regional founder effects. This particular component of the demographic history of the French-Canadian population has resulted in a geographic heterogeneity of genetic diseases in Quebec, with more than twenty Mendelian diseases occurring at unexpectedly high frequencies in some areas of the province [19], [22].

Here, we specifically test the theory that deleterious mutations accumulate and/or persist in a population that has undergone a demographic bottleneck and rapid expansion in a short period of time, potentially as a consequence of reduced selection, using the French-Canadian population of Quebec. It has been argued that colonists at the forefront of expansions have a fitness advantage [23]. Here we show that if this is the case, then this short-term fitness advantage may come at an overall long-term cost. We also aim to describe how this complex demographic scenario has shaped the genetic variation in a modern population; as of yet, no study has described how the original genetic bottleneck and subsequent population expansion have affected the full-spectrum of genetic variation among French-Canadians.

## Results

Through exome-sequencing, we set out to determine how the distribution of variants in a founder population differs both in overall frequency, and potential functional impact relative to the source or progenitor population. All major observations were replicated on two different sequencing platforms and with similar sample sizes (see Material and Methods and results below). In total, we detect 64,631 high-quality SNPs in 109 individuals from the French-Canadian population with low error rates (see Material and Methods). Using previously described data [24], we find a total of 46,662 high-quality SNPs from 30 individuals in the French population. The difference in the number of SNPs detected is largely driven by the different sample sizes. The numbers of SNPs falling into each functional category are shown in Table S1. Compared to French individuals, French-Canadians have lower levels of heterozygosity (on average 19.2% and 11.5% of the variants per individual are heterozygous in French and French-Canadians, respectively) and have lower average nucleotide pairwise diversity (Table 1). Reduced genetic diversity in the French-Canadian population is consistent with the historically documented population bottleneck.

**Table 1:** Population genetic measures in the 38-Canadian populations.

	Sample size	Coding SNPs	*θ_W_ (×10^−4^)	π (×10^−4^)	D†	Het‡
French	30	32,187	3.50	2.97	−0.14	19.2%
French-Canadian	109	44,485	2.77	1.81	−0.41	11.5%

* Watterson's estimate.

† Tajima's D, calculated as an average across genes with five or more segregating sites.

‡ Heterozygosity, calculated as the percentage of heterozygous variants per individual at variant sites.

**Figure 1:**
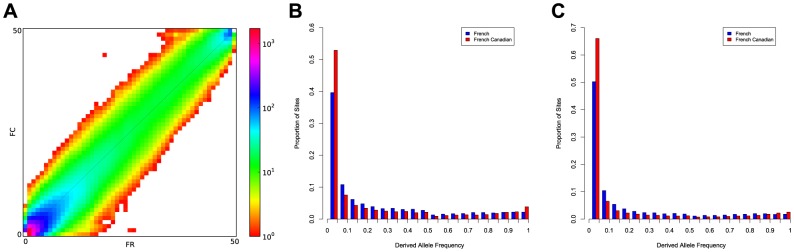
Site frequency spectrum in the French and French-Canadian populations. A) Joint frequency spectrum of genetic variation between the French (FR) and the French-Canadian (FC) populations, projected to 50 samples per population; B) Site frequency spectrum in the French and the French-Canadian population for the synonymous variants using derived allele frequency; C) Site frequency spectrum in the French and the French-Canadian population for the missense variants using derived allele frequency.

The French-Canadian population also exhibits an excess of low frequency variants in comparison to the French population (Figure 1), and the proportion of variants with MAF<5% is significantly higher in the French-Canadian population (p<0.01). The excess is not a consequence of different sample sizes; if we resample the same number of individuals from each population and include only sites where all individuals pass identical quality filters, we observe a similar excess of rare variants in the French-Canadian population compared to the French population (Figure S1). The distribution of allele frequencies is likely indicative of the population expansion undergone by French-Canadians after the bottleneck out of Europe and is supported by lower per locus Tajima's D values (Table 1) when compared to the French population (t-test p value = 6.51e-15) (Figure S2). As seen in previous studies, low frequency classes are enriched for nonsense and missense variants in relation to synonymous variants (Figure S3). Strikingly, among the total number of SNPs, only a relatively small fraction (36.5%) are shared between the two populations (Figure S4) and this fraction decreases for functional SNPs (missense, nonsense, splice site), which are enriched for rare variants. When considering those variants shared between populations, we find a high level of agreement; of the 29,767 variants shared by both populations, the vast majority have extremely low FST scores (97.6% are less than 0.05), indicating little population differentiation for most common variants.

In order to compare the French-Canadian SNPs to a larger dataset, we extended the comparison to a list of variants discovered from high-coverage sequencing of exomes in 85 CEU individuals in the 1000 Genomes Project [9], as well as 1,007 individuals from other populations from the same resource, and find that the French-Canadian population shows a high percentage of private variants not found in any other population (Table 2). The distribution of these non-shared variants is asymmetric, and is enriched for rare and missense variants. The proportion of private variants is lower than those reported in comparisons across different continents, but higher than proportions observed across populations in the same continent [12], [13]. Roughly, populations in different continents share only about 10% of rare variants, while close populations in the same continent, such as individuals from the CEU and Tuscany (Italy) populations, share about 90% of rare variants [7], [8], [12], [13].

**Table 2:** Shared SNPs between the French-Canadian population and the 1000 Genomes Project populations.

	Rare (MAF≤2%)	Rare (MAF≤5%)	Common (MAF≥20%)	Total
Total SNPs	30,193	39,323	12,223	64,630
CEU population (%)	37%	47%	99%	67%
All populations (%)	62%	68%	99%	80%
Nonsynonymous SNPs	13,541	16,760	3,453	24,418
CEU population (%)	33%	41%	98%	58%
All populations (%)	56%	61%	98%	72%
Synonymous SNPs	8,477	11,403	4,368	20,067
CEU population (%)	40%	52%	99%	72%
All populations (%)	67%	73%	99%	84%

**Figure 2:**
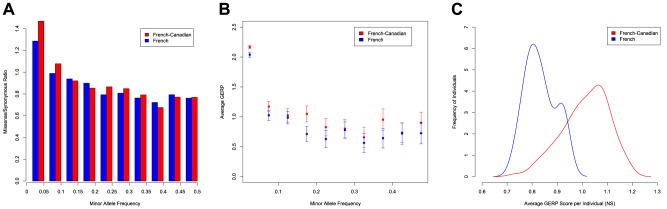
Excess of functional variants in the French-Canadian population. A) Ratio of nonsynonymous to synonymous changes in the French and the French-Canadian populations for variants grouped by minor allele frequency. B) Average GERP value of the functional changes for each frequency class in the French and the French-Canadian populations. C) Distributions of the average GERP scores at functional sites per individual in the French and the French-Canadian populations. GERP scores are averaged per individual by using only sites at which each individual carries the minor allele.

Given that we observe an excess of rare variants at functional sites in the French-Canadian population, we consider the effect of these variants on fitness and selection using a number of different approaches. First, we test for differences in the ratio of missense to synonymous changes within the SFS (Figure 2). Whilst the missense to synonymous ratio in the French population for SNPs with MAF<5% (1.31) is very similar to that observed in other populations [11], the French-Canadian ratio of 1.47, points to a major fraction of deleterious SNPs in the population, which carries a significantly larger proportion of rare mutations at missense sites (p<0.01, chi-squared test). For the most common variants (MAF>0.25), the French and French-Canadian populations have identical missense to synonymous ratios (0.77). Second, we consider the predicted effects of nonsynonymous variants using GERP scores [25] and find more evidence for an excess of potentially damaging mutations in the French-Canadian population. GERP is a measure of conservation that is calculated across 34 mammalian species [25] and since it inversely correlates with derived allele frequency (DAF) [26], [27], it can be used to classify genetic variants and is often used as part of a criteria to prioritize functional variants in disease studies [28]. Comparing missense and nonsense SNPs in the French and French-Canadian populations, we find that the average GERP score is significantly higher for mutations in the French-Canadian population (Wilcoxon signed-rank test, p = 0.004). The difference is particularly strong for SNPs at the lowest frequencies (Figure 2), which are enriched for mutations with a higher impact on protein function, but the average GERP score for variants with MAF>10% is also significantly higher in the French-Canadian population (p<0.01). Conversely, we do not observe significant differences between populations when synonymous changes are compared (Wilcoxon signed-rank test, p = 0.846). The same enrichment for higher GERP scores in the French-Canadian population is also seen when comparing the distribution of average GERP scores for alleles carried at missense sites within each individual (Figure 2), and overall French-Canadian individuals have significantly higher mean GERP scores than French Individuals (Wilcox-rank sum test, p<0.001). Third, a significantly higher proportion of missense variants are predicted to be damaging in the French-Canadians compared to the French population using Polyphen [29] (49.5% and 45.5% respectively, p<0.01), indicating that on average variants segregating in the French-Canadian population tend to be putatively more damaging.

The inference of a higher proportion of deleterious alleles in the French-Canadian population is not a consequence of different sample sizes; resampling thirty individuals from the French- Canadian population, we again find a significantly larger missense to synonymous ratio for rare alleles (1.44, p<0.01), a significantly higher average GERP score for rare alleles at missense sites (2.194 for resampled French-Canadians, 2.067 for French, p<0.01) and a significantly larger proportion of missense variants predicted to be damaging by Polyphen (49.6%, p<0.01) for French-Canadians when compared to the French population. Furthermore, it is unlikely that the excess of rare deleterious alleles in French-Canadians is driven by data quality since we estimate a false positive rate of ∼0.2% for singletons (see Material and Methods), which are most likely to be enriched for error.

To understand why there is an excess of putative damaging variants in the French-Canadian population, we analyzed the intensity of natural selection in both the French and French-Canadian populations. First, we estimated the demographic parameters and the population selection parameter (γ = *Ne(s)*) using the Poisson Random Fields method implemented in *prfreq* [14]. To estimate population demographic parameters, we used synonymous sites to test different demographic models and we find a significantly better fit for models that include a bottleneck and expansion compared to neutral stationary models for both the French and the French-Canadian populations (Tables S2 and S3, Kolmogorov-Smirnov (KS) tests, p>0.05 in both cases). Although these models are necessarily simplified to capture key demographic processes rather than a literal history of the populations, we used them as a correction factor when next attempting to infer selection parameters at nonsynonymous sites. To this end, models including both the three-parameter demographic history and negative selection have a significantly better fit to the observed site frequency spectrums for both the French and French-Canadian populations at nonsynonymous sites than models assuming neutrality or including demographic history alone (Tables S2 and S3 and Figure S5, p<0.001). As expected, including selection does not significantly improve the fit to the site frequency spectrum at synonymous sites, which provides a good check on the demographic model. The estimated γ parameter in French-Canadians is substantially less negative than that in the French population (γ = −115 in French population, γ = −82 in French-Canadian population, p<0.001), which could be at least partially a result of smaller *Ne* in the French-Canadian population.

Second, we estimated the distribution of fitness effects (DFE) of mutations segregating in French and French-Canadian populations using the DFE-alpha software (http://homepages.ed.ac.uk/eang33/), which predicts the effects of new deleterious mutations using the site frequency spectrum [30]. The DFE estimated for the French population is broadly similar to that predicted for the European population in a previous study [31] using the two epoch model (Table 3), and the mean selective effect (*Ne*(s)) is similar to the γ value predicted by *prfreq*. Interestingly, the DFE estimated for the French-Canadian population has a much lower mean selective effect for new deleterious mutations of 12.8 (compared to 104.9 for the French population). Furthermore, the proportion of strongly selected deleterious mutations is much lower in the French-Canadian population compared to the French (Table 3), which could reflect a relaxation of selection in the French-Canadian population due either to a reduction in *Ne* or the new environment, that has subsequently led to an accumulation or the persistence of potentially harmful rare variants.

**Table 3:** Estimated parameters for the distribution of fitness effects of new deleterious mutations.

Population	*NeE(s)* (95% CI)	β (95% CI)	*Nes* (95% CI)			
			0–1	1–10	10–100	>100
French Canadian	13 (5,29)	0.24 (0.15,0.42)	0.42 (0.36,0.49)	0.29 (0.20,0.44)	0.27 (0.14,0.29)	0.02 (0.0,0.09)
French	105 (61,204)	0.15 (0.12,0.17)	0.40 (0.38,0.44)	0.16 (0.14,0.19)	0.22 (0.18,0.25)	0.21 (0.16,0.26)
European (EPG)	61 (16,4.8×10^5^)	0.29 (0.08,0.54)	0.23 (0.15,0.33)	0.22 (0.06,0.33)	0.36 (0.07,0.48)	0.19 (0.01,0.54)
European (PGA)	51(16,->∞)	0.19 (0.04,0.32)	0.37(0.29,0.46)	0.20 (0.04,0.31)	0.27 (0.04,0.35)	0.15 (0.02,0.45)

European results are taken from Keightley *et al* [31]. The table shows estimates for the mean selective effect (*NeE(s)*), the shape parameter of the distribution of selective effects (β) and the proportions of mutations falling into each group of selective effects.

Finally, to test whether the differences we observe between the two populations are driven by different sequencing platforms, we analyzed data from an additional 50 French-Canadian individuals sequenced on Illumina's HiSeq platform and compared the results to the French dataset; we replicate all of the major findings. First, we observe a significant excess of rare variants in the French-Canadian Illumina dataset compared to the French (57.4% and 45.3% of variants with MAF≤5% respectively, p<0.01, Figure S6, Table S4). Similarly, comparing datasets sequenced on the SOLiD platform by considering a further European dataset (CEU population from the 1000 Genomes Project), we again find an excess of rare variants in the French-Canadian population (p<0.01, Figure S6). Second, we find a significantly larger missense to synonymous ratio for rare alleles (1.39, p<0.01, Table S4) and a significantly larger proportion of missense variants predicted to be damaging by Polyphen (48.2%, p<0.01, Table S4) for the French-Canadian Illumina dataset compared to the French. Finally, rare alleles at missense sites have a significantly larger GERP score on average in the French-Canadian Illumina data (2.194, p<0.01, Table S4) when compared to the French population and when considering the distribution of average GERP scores at missense sites within these individuals, French-Canadians have significantly higher mean GERP scores than French Individuals (p<0.01, Figure S7).

## Discussion

Recent deep resequencing of human populations has highlighted an accumulation of rare variants above that expected under Wright-Fisher models [3]–[8]. Using exome resequencing data from over a hundred French-Canadian individuals, we show that a human founding population that has undergone rapid expansion contains an excess of private and rare variants compared to the French population after a colonization event less than 20 generations ago. Genetic variants in French-Canadians tend to be putatively more deleterious than those in the French. On the population level, evidence for this comes from the fact that mutations in the French-Canadian population tend to occur at functional sites with higher conservation scores and/or sites predicted to be damaging, are located preferentially at missense sites, and have higher missense to synonymous ratios than in French and European populations. Furthermore, at the individual level, this potentially translates into an increased genetic burden, since although French-Canadians carry a similar number of derived alleles as the French, these alleles tend to occur at more putatively damaging sites, as indicated by alleles in French-Canadians occurring at sites that on average have higher GERP scores (Figure 1C). Furthermore, since the French-Canadian population shows lower levels of heterozygosity (and thus higher levels of homozygosity), this may have implications for disease susceptibility.

It is known that the incidence of around twenty Mendelian diseases is higher in Quebec [19], [22] and some hereditary diseases show a particular pattern in the French-Canadian population, with local enrichments within particular geographical areas originated by regional founder effects [19], [22], [32]. Although it is difficult to translate our results into specific population genetic risk estimates, it may be possible that the increase of rare deleterious variants and reduced heterozygosity in the French-Canadian population is leading to higher disease risk. Rare alleles that were present at damaging sites in the original population may subsequently have been removed in the French population, yet still persist in French-Canadian individuals due to sampling effects, smaller population sizes, less competition and a higher birth rate. Although this seems unlikely to impact upon diseases caused by recessive variants in homozygous form, damaging mutations that have arisen since the founder event may be dominant or serve as the second, and ultimately vital, mutation within an important gene under a compound heterozygous model of Mendelian disorders. Furthermore, we find some evidence that higher frequency variants (MAF>10%) are on average more damaging in the French-Canadian population when compared to the French, since they tend to have higher GERP scores (see Results), which may impact upon the incidence of Mendelian diseases under a homozygous recessive model.

It has previously been shown that there is proportionally more deleterious variation in European populations after the out of Africa expansion [14], [15]. However, this process occurred over a much longer timeframe and also relies on a long bottleneck to explain the increase in deleterious variants in Europeans [14], [15]. In French-Canadians we observe a similar increase of rare deleterious variants but over a markedly short time frame. Furthermore, since the French-Canadian population did not undergo a long population bottleneck, the excess of deleterious variants could be explained by a rapid expansion of the population as well as other demographic factors such as subsequent regional founder effects in Quebec. To test this we performed a number of forward simulations incorporating selection and the demographic history of Europe, as inferred in a recent study [14], followed by a simple population bottleneck and rapid expansion in the French-Canadian population, and a less extreme expansion in the French population (for details, see Materials and Methods). We modeled population bottlenecks of varying sizes, performing 100 replicates for each scenario, and then calculated the difference in the proportion of variants with MAF<5% between the French and French-Canadian populations (Table S6). The scenarios modeled likely represent a simplified version of the actual demographic history of the French and French-Canadian populations, however we use them here to test differences between populations undergoing different rates of expansion under selective constraint after sharing a large proportion of demographic history. Under these models, the largest increase in rare variants in French-Canadians occurs when the population did not undergo a bottleneck, showing differences as large as 5.23% across the 100 replicates, with an average shift of 1.09%. Furthermore, we also observe on average an additional 8.32 deleterious alleles per megabase (defined as having a negative selection coefficient) per replicate segregating in the French-Canadian population compared to the French population. For simulations including a bottleneck, the biggest increase of rare variants in the French-Canadian population occurs for a bottleneck of 75%, with differences as large as 5.74% across the 100 replicates, an average increase of 0.74% and an additional 5.86 deleterious alleles per megabase per replicate in the French-Canadian population.

Although these simple models lead to an increase in the proportion of rare variants in the French-Canadian population, the shift observed in the empirical data, which shows an increase of 9.8% of variants with MAF<5% in the French-Canadian population compared to the French population when using the same sample sizes (see above and Figure S1), is larger than that generated by simulations; there are several possible explanations for this. First, it may be that current tools are not able to accurately model recent events such as a rapid population expansion. Second, it is likely that a more complex demographic scenario is needed to explain the size of the increase in rare variants in the French-Canadian population, that may also include changes in selective forces as a consequence of the reduced competition occurring between a small number of founders. In fact, the French-Canadian population is genetically stratified into subpopulations with differentiated demographic histories [19], [21], [22]. Independent settlements and expansions with partially reduced genetic exchange across subpopulations, unequal contribution to the current genetic pool, as well as some admixture with other populations could have also contributed to the shift in the site frequency spectrum. Consistent with these notions, a recent study focusing on a specific sub-founding population within Quebec presented evidence that individuals on the wave front of colonization events have a heritable advantage and a higher contribution to the current genetic pool [23]. In this study, we have not focused on specific regions within the population and have not tested this observation. However, our results do demonstrate that the recent founding event and subsequent colonization events may have had a substantial deleterious impact across genomes. To a lesser extent, rare variants could also arise from the inclusion of founders from different regions in France or other European countries, which could be also related to the level of genetic diversity in Quebec, similar to that reported for European populations [21]. Similarly, the unequal sex ratio of the Quebec settlers of more than ten times more men than women [18], may also have contributed to a shift in the effective population size and loss of heterozygosity. Finally, although there is evidence of a population bottleneck in the French-Canadian population, such as reduced levels of heterozygosity, given the results of our simulations it seems unlikely that the bottleneck was particularly strong.

In this study, we show that even in the case of two very close populations that are separated by only 400 years approximately, the differences in the landscape of genetic variation can be substantial under particular demographic conditions. Rare variants are presumed to explain some of the missing heritability not accounted for by common variants in genome wide association analyses for complex disorders [33] as well as most of the rare diseases. Furthermore, there is mounting evidence that coding rare variants are contributing to complex traits [34]. The high number of population private rare functional variants described in this study constitutes a challenge for genetic association studies, affecting the replicability and correlation of genetic risk factors across human populations. Indeed, even from a relatively limited number of French-Canadian chromosomes, we discovered a substantial number of missense mutations that are not found on the widely used Illumina exome-arrays built from SNPs ascertained across a number of major sequencing studies. One third of the missense SNPs we discovered from sequencing over one hundred exomes are not found on these arrays, variation that likely influences complex traits and disease phenotypes, but is missing from analysis of disease risk. Although we understand from population genetics that most variants will be rare, this observation speaks to the need for continued sequencing of isolated or semi-isolated populations. Beyond the particular case of the French-Canadian population, this study highlights the importance of local demographic events in shaping genetic variation, and the need for creating population-based catalogues of human genetic variation [12].

## Materials and Methods

### Ethics statement

This research has been approved by the CHU Sainte-Justine's ethical committee. Data was analyzed anonymously.

### Samples

One hundred and fourteen French-Canadians were selected for sequencing. French-Canadian samples are the healthy parents of four disease cohorts (primary immunodeficiencies, acute lymphoblastic leukemia, schizophrenia and autistic spectrum disorder) recruited at the Sainte-Justine Hospital (Montreal). Additionally, sequences from 30 French samples previously analyzed were included in the study [24]. We used principal component analysis to identify and remove the genetic outliers (see below).

### Exome capture and sequencing

Exome capture was performed with the SureSelect Target Enrichment System from Agilent Technologies optimized for Applied Biosystems SOLiD sequencing, using the Agilent SureSelect All Exome Kit (38 Mb) and the Human All Exon 50 Mb kit covering exons annotated in the consensus CCDS [35]. Analyses were performed considering the coding regions targeted in the Agilent SureSelect All Exome Kit (38 Mb). Briefly, 3–5 µg of DNA were sheared by sonication, 5′ ends repaired, and the resulting fragments were ligated to adaptors, which were then run in size-select gels to select fragments of 150–250 bp in size. The extracted DNA was amplified by PCR and hybridized to the capture library containing the human exome. Hybridization was performed in a solution at 65°C for a minimum of 24 hours, followed by washing and capture of the hybridized DNA through magnetic bead selection, PCR and purification. Quantification of DNA libraries was performed using a Bioanalyzer and qPCR instrument. Exome sequencing was performed using SOLiD 3 Plus and SOLiD 4 Systems (Applied Biosystems), following the manufacturer's recommended protocols. Sequence reads were aligned to the human genome reference sequence (hg18, downloaded from http://genome.ucsc.edu) with BioScope, the available mapping tool for the SOLiD technology. GATK recalibration [36] was applied after mapping, PCR duplicates removed with Picard (http://picard.sourceforge.net) and SNP calling was performed using Samtools [37]. In total, 61 gigabases of sequencing reads mapped to the reference genome, with an average of 86% of the targeted regions being covered by at least one sequencing read. Each individual had an average coverage of 17-fold (see supplementary material, Table S5). SNP annotation was performed using the SeattleSeq Annotation tool (http://gvs.gs.washington.edu/SeattleSeqAnnotation/). Variants from the French population were generated from exome sequencing of the same targeted exons using Illumina sequencing [24].

### High quality dataset

Stringent variant calling criteria were applied to produce a high quality dataset of both the French and French-Canadian populations, including only variants that satisfy all of the following conditions: (i) fall within the regions targeted by the Agilent SureSelect exome capture kit, (ii) with SNP consensus or variant quality of 30 or higher, (iii) with sequence coverage of 10-fold depth or greater and (iv) in Hardy-Weinberg equilibrium (using a stringent p-value of 0.001). Furthermore, variants were included only if these criteria were met in at least 20 individuals in both the French and French-Canadian populations. The average transition/transversion ratio for all the French and French-Canadian samples in the coding variants was 3.32, as expected for exonic sequences [38] and we detected no significant difference between French and French-Canadian samples (3.38 and 3.30, respectively). Similarly, frequencies of the twelve possible nucleotide changes are similar between the two populations (Figure S8). For the resampling analyses, we randomly choose thirty individuals from the French-Canadian population and applied the same filters as above.

### Population structure analysis

In order to use the most genetically homogeneous group of individuals in each population we performed principal component analysis (PCA) for each population sample using SmartPCA as implemented in the program eigenstrat [39]. First, PCA was performed within each population including variants called in at least 80% of the individuals in each population to avoid the effects of missing values; these variants totaled 13,035 positions for the French-Canadian population and 26,843 for the French population. Significant PCs were inferred using the TW-statistic (p-value<0.01) and outlier individuals were identified based on their individual loading exceeding two standard deviations from the mean of each significant axis. This analysis revealed five outlier individuals in the French-Canadian population and none for the French samples (Figure S9). Removing outlier individuals based on population structure analysis of each population separately resulted in the retention of 109 French-Canadian and 30 French individuals for subsequent analyses. Next, we performed PCA combining both populations, including only positions called in at least 80% of the combined samples, and only individuals with missing data less than 1%. This represented a total of 4,588 SNPs in 89 samples. We find no obvious differences between the two populations (Figure S9), although the French-Canadian population seems to show a slightly lower level of diversity and represents only a subset of the total genetic variation in the French population. The joint frequency spectrum of genetic variation was represented using the δaδi software [40].

### SNP validation

We performed a number of validation procedures to check the quality of our data. First, we performed Sanger sequencing on a total of 113 heterozygous calls detected in the individuals included in this study (89% of the 97 variants have MAF<5% and 54% were singletons). In total we confirmed 109 calls, giving a false positive rate of 3.5%. This figure probably represents an upper bound, since the variants selected for validation are enriched for rare variants which are known to be more prone to sequencing errors [41]. Second, we sequenced the offspring of 16 individuals from the French-Canadian population, using the same protocols and filtering steps as in the parents, in order to confirm the presence of certain alleles in the population. Thus, to check the false positive rate for variants that are likely to contain the most errors (singletons), we isolated any positions in the parents that were singletons in our population and then checked to see if the variant is called in the child, only including the position if the same quality filters were met in the offspring (variant quality>30, coverage>10). Under normal patterns of Mendelian inheritance we expect 50% of singletons to be inherited by the child. Overall, we observe 4,666 singletons across the 16 individuals, 2,328 of which are present in the offspring (49.89%), representing a false positive rate for singletons of ∼0.2%.

Third, we also tested the quality of our data by comparing DNA and RNA sequences for three French-Canadian individuals using the same high quality filtering criteria in both datasets (consensus or variant quality greater than 30, coverage greater than 10). For RNA sequencing, RNA was enzymatically fragmented, and cDNA generated by reverse transcription from adaptors ligated to ends of the RNA molecule. Then, the cDNA was amplified using primers complementary to adaptors and purified. Sequencing was performed in a single SOLiD slide containing barcoded samples. Sequence reads were aligned to the human genome reference sequence (hg18, downloaded from http://genome.ucsc.edu) with SOLiD's BioScope mapping tool. Recalibration was performed with GATK [36], and PCR duplicates were removed with Picard (http://picard.sourceforge.net). SNP calling was performed using Samtools [37]. As differences may exist between DNA and RNA as a consequence of RNA editing [42]–[45] and allelic expression [46], for positions that are heterozygous in DNA, we considered a site as successfully validated if at least one read was present in RNA for both alleles; we confirm 474 of 506 sites. Since it is known that approximately 28% of genes show greater than a 4-fold difference in the expression of two alleles in RNA [46], it is likely that some differences between DNA and RNA are driven by allelic specific expression. Indeed, 5 out of the 32 sites that fail validation in one individual show evidence for being heterozygote (displaying at least one read from each allele) in the RNA of at least one of the other two individuals that were sequenced. Differences between DNA and RNA at heterozygous sites are not significantly enriched for rare variants; only 5 out of 32 sites that fail validation have MAF<5% (variants with MAF<5%, 5/66 not validated, p = 0.92). Furthermore, we also considered sites that contained homozygous non-reference alleles in DNA sequences and then checked the corresponding position in RNA. All 242 positions were validated, further confirming the quality of the data.

Finally, to consider the quality of common variants, we compared the genotype frequencies at polymorphic sites obtained from our exome sequencing that overlapped with data from 521 French-Canadian individuals that were genotyped on Illumina's Omni 2.5M arrays. In each case we compared the number of homozygous reference, homozygous alternative and heterozygous calls in our exome data with the same number of randomly sampled individuals from the chip data. In total, 23,231 sites were overlapping, 99.94% of which were not significantly different between exome sequencing and array data (p>0.05, after Bonferroni correction).

### Selection coefficient and fitness effect analysis

To estimate the strength of purifying selection in the French and French-Canadian populations we applied two methods. First, we used *prfreq*, a program that uses Poisson random fields [14] to estimate the maximum likelihood values for different scenarios given an observed site frequency spectrum (SFS). For the French and French-Canadian populations, we projected the SFS down to 60 alleles by randomly sampling individuals from the French-Canadian population and including only sites with 0% missing data. The ancestral allele was inferred from the homologous chimpanzee sequence obtained from Seattleseq annotation (http://gvs.gs.washington.edu/SeattleSeqAnnotation/) and since mutation rates vary across the genome as a function of neighbouring nucleotides [47], we corrected for the uncertainty of the ancestral sequence following the method of Hernandez *et al* [48]. Maximum likelihood values for each scenario were obtained with a multinomial calculation that estimates the probability of each SNP segregating at a given derived allele frequency. P-values associated with various demographic and selective models were estimated using likelihood-ratio tests. Demographic parameters were inferred from the site frequency spectrum of synonymous variants comparing three scenarios: a stationary population, contraction/expansion, and a population bottleneck and expansion (Tables S2 and S3). Finally, the selective parameters were obtained by comparing the likelihood of the missense SFS using the demographic model inferred from synonymous variants (see above) to the likelihood for the same demographic model incorporating a selection parameter (*γ* = 2*Ne(s)*). To compare the *γ* values estimated in the French and French-Canadian populations we compared the likelihoods estimated in each case with the likelihoods computed using the *γ* values from the other population.

Second, to calculate the distribution of fitness effects associated with mutations occurring in the French and French-Canadian populations we used the DFE-alpha software [31] (http://homepages.ed.ac.uk/eang33/). To construct the unfolded site frequency spectrums for the two populations we included variants and sites in the targeted region in which at least 30 and 90 individuals passed the high quality filters for the French and French-Canadian populations respectively. These numbers were chosen to reduce the amount of missing data at each site, whilst retaining the majority of polymorphic sites for analysis. We then counted the number of sites that had zero to 180 derived alleles in the French-Canadian population, where derived alleles represent sites that have diverged from chimpanzee. The same approach was applied for the French population using 60 chromosomes. For the French-Canadian population, ninety individuals were sampled randomly without replacement at sites where the number of alleles passing quality filters exceeded 180. Derived alleles were inferred from chimpanzee sequences and human and chimpanzee pairwise alignments were downloaded from the UCSC website (http://hgdownload.cse.ucsc.edu/downloads). As in the original DFE analysis [31], intronic sites served as the neutral standard, the distribution of fitness effects was fit to zero-fold degenerate sites and any sites that were part of a CpG dinucleotide were removed. Confidence intervals were generated by bootstrapping; sites were selected randomly across the site frequency spectrum with replacement to generate 100 new datasets for each population.

### Replication datasets

To replicate the major findings of this study we analyzed data from a cohort of fifty French-Canadian individuals sequenced on the Illumina platform representing the unaffected parents from different disease projects (developmental delay and fetal malformations). Exomes were captured from 3 µg of blood genomic DNA, using the Agilent SureSelect Human All Exon Capture kit (V3 and V4; Mississauga, ON), and sequenced paired end using the Illumina Hi2000seq technology. Raw sequencing data was processed using the same pipeline and filtering process as described above, including only those sites that are sequenced in all datasets. PCA was performed as before, taking SNPs with MAF>5% and missing data<5% - zero outliers were removed (Figure S10). For the CEU population, we obtained BAM files for 35 individuals from the 1000 Genomes Project ftp site (ftp://ftp-trace.ncbi.nih.gov/1000genomes/ftp/) sequenced on the SOLiD platform and applied the same pipeline and filters as detailed above.

### Simulations

To test for an increase in rare variants in the French-Canadian population we simulated a number of demographic scenarios under selection using the forward simulator SFS_code [49]. First, we implemented timing and population size scaling for the European demographic history, as detailed in the SFS_code documentation (http://sfscode.sourceforge.net/SFS_CODE_doc.pdf, figure 2, model taken from [14]). This model includes an initial burn-in period with a population size of 7,895, followed by a bottleneck at time zero to a population size of 5,699. Following this, the population remains at constant size for 7,703 generations before an instantaneous growth to 30,030, which remains for a further 874 generations. We scaled this model using an ancestral population size of 1,000. Then, we simulated a population split and a bottleneck of 50%, 75% and 100% (no bottleneck) for one of the populations to represent the founding of Quebec, scaled using the initial population size to occur twenty generations ago. This was then followed by exponential growth over twenty generations in the European and Quebec populations to increase their size by 3 and 600 respectively (as documented in historical records), using 100 replicates for each scenario. In total, we simulated 360 unlinked genes per replicate, each consisting of five 400 bp exons separated by introns of size 2 kb (similar to the average exon and intron sizes documented in [50]). We used a mutation rate per site of 1.5×10^−8^ and an average recombination rate of half this value. We ignored positive selection since it is likely to be rare and used an average selection coefficient of −0.03, as inferred in [14], sampled from a gamma distribution. In each replicate and for each population, we selected 100 individuals and then compared the proportion of variants with MAF<5%. The results of these simulations are shown in Table S6.

## Supporting Information

Figure S1 Site frequency spectrum for French and French-Canadian populations using the same sample size. Thirty individuals were selected at random from the French-Canadian population and for both populations only sites with no missing data are considered.
(PDF)

Figure S2 Tajima's D values in the French and the French-Canadian populations. Each dot represents the value for each gene and the least squares regression line is shown in red.
(PDF)

Figure S3 Site frequency spectrum for the synonymous, missense and nonsense variants in the French-Canadian population.
(PDF)

Figure S4 Percentage of shared and private variants between the French (N = 30) and the French-Canadian populations (N = 109) in this study.
(PDF)

Figure S5 Observed and expected folded site frequency spectrum using *prfreq*. Observed and expected site frequency distributions for the synonymous SNPs in the French population (A), synonymous SNPs in the French-Canadian population (B), nonsynonymous SNPs in the French Population (C), and nonsynonymous SNPs in the French-Canadian population (D). Expected distributions have been obtained with a neutral model not including demography, a model with demography, and for the nonsynonymous variants a model with demography and selection is also included (see parameters in Table S3).
(PDF)

Figure S6 A) Site frequency spectrum in the French and the French-Canadian populations using Illumina datasets for the synonymous variants; B) Site frequency spectrum in the French and the French-Canadian populations using Illumina datasets for the missense variants; C) Site frequency spectrum in the CEU and the French-Canadian populations using SOLiD datasets for the synonymous variants; D) Site frequency spectrum in the CEU and the French-Canadian populations using SOLiD datasets for the missense variants.
(PDF)

Figure S7 Distributions of the average GERP scores at functional sites per individual in the French and the French-Canadian populations. GERP scores are averaged per individual by using only sites at which each individual carries the minor allele.
(PDF)

Figure S8 The proportions of the twelve possible nucleotide changes in each population.
(PDF)

Figure S9 Principal Component Analysis with A) the 114 French-Canadian samples; B) the 109 French-Canadian samples after removing five genetic outliers; C) the 30 French samples (no genetic outliers were detected); D) the 109 French-Canadian and the 30 French samples.
(PDF)

Figure S10 Principal Component Analysis of the 50 French Canadians sequenced using the Illumina technology.
(PDF)

Table S1 Total number of SNPs in the 38 Mb targeted regions in the French and the French-Canadian populations.
(DOCX)

Table S2 *prfreq* maximum likelihood estimates of neutral, demographic and selective models for the French Population.
(DOCX)

Table S3 *prfreq* maximum likelihood estimates of neutral, demographic and selective models for the French Population.
(DOCX)

Table S4 Summary values for tests for rare and damaging variants in the French and French-Canadian populations. Rare variants are defined as those with a minor allele frequency less that 5%.
(DOCX)

Table S5 Sequencing statistics for the 144 samples included in this study.
(DOCX)

Table S6 Results from forward simulations modeling the demographic histories of the French and French Canadian (FC) populations.
(DOCX)
