# Correction: Whole-Exome Sequencing Reveals a Rapid Change in the Frequency of Rare Functional Variants in a Founding Population of Humans

**DOI:** 10.1371/journal.pgen.1004260

**Published:** 2014-02-28

**Authors:** The PLOS Genetics Staff

The seventh author's name is spelled incorrectly. The correct name is: Jean-Christophe Grenier.
