# Supplementary figures and images for: Whole-Exome Sequencing Reveals a Rapid Change in the Frequency of Rare Functional Variants in a Founding Population of Humans
Source: PLoS Genet. 2013 Sep 26;9(9):e1003815. doi: 10.1371/journal.pgen.1003815 (PMC3784517; doi:10.1371/journal.pgen.1003815)

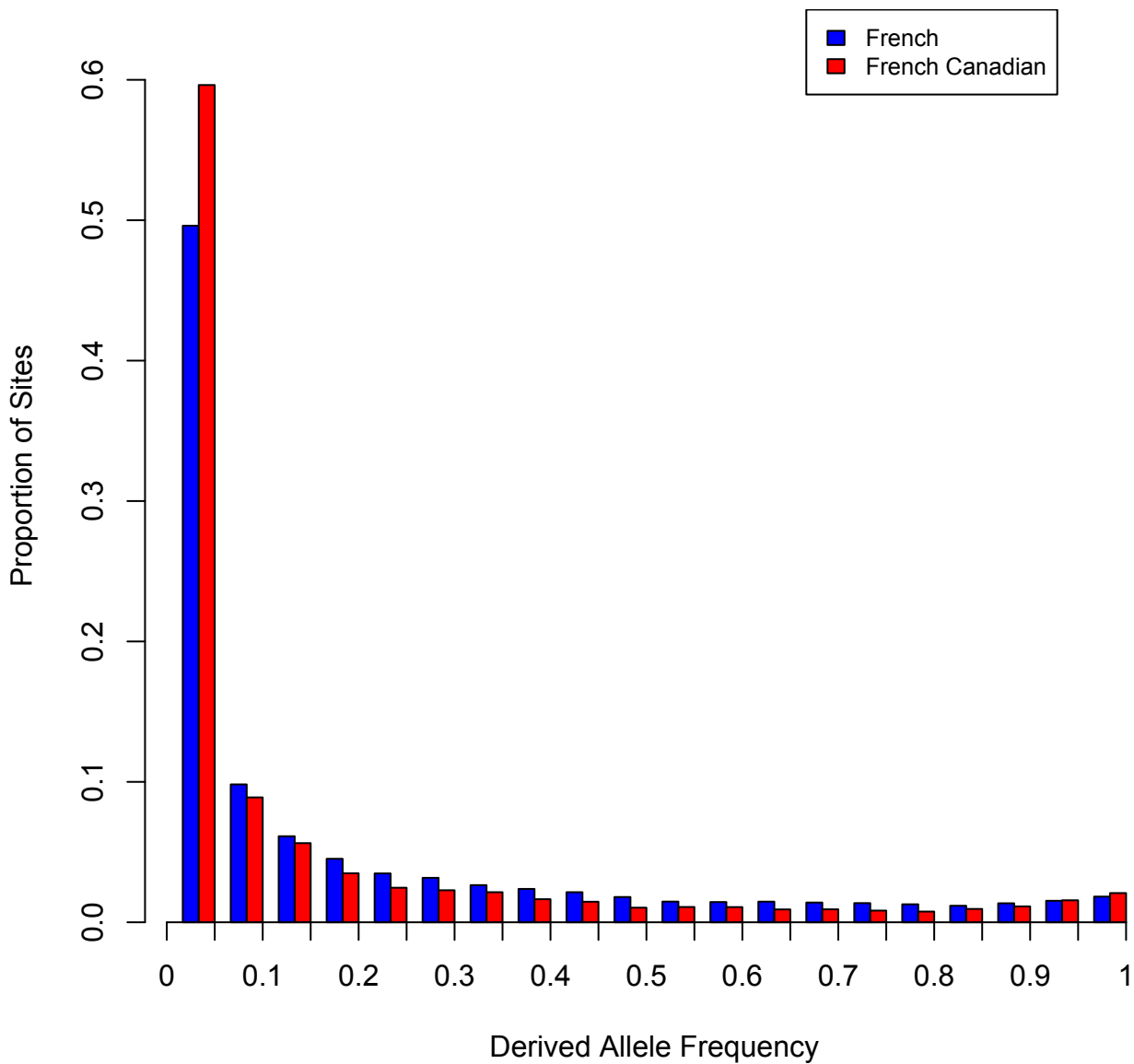

Supplement: Figure S1 — Site frequency spectrum for French and French-Canadian populations using the same sample size. Thirty individuals were selected at random from the French-Canadian population and for both populations only sites with no missing data are considered. (PDF) [file pgen.1003815.s001.pdf]

French Canadian Tajima's D per locus

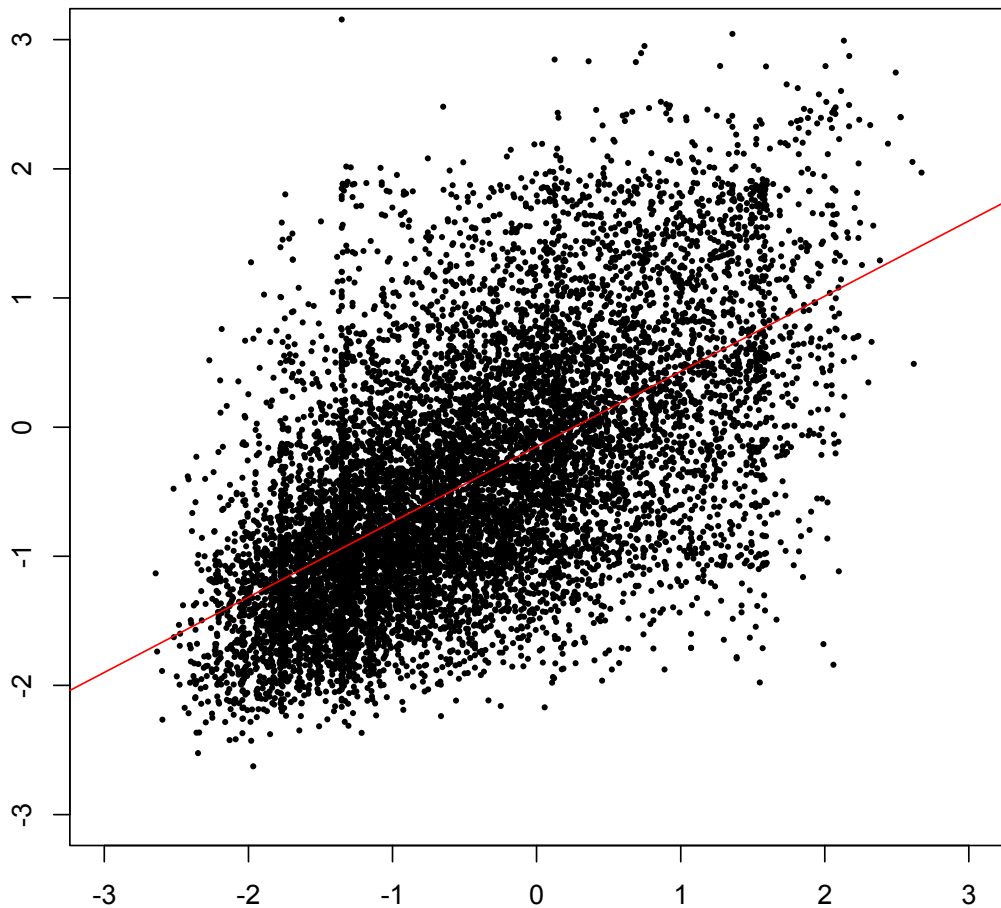

French Tajima's D per locus

Supplement: Figure S2 — Tajima's D values in the French and the French-Canadian populations. Each dot represents the value for each gene and the least squares regression line is shown in red. (PDF) [file pgen.1003815.s002.pdf]

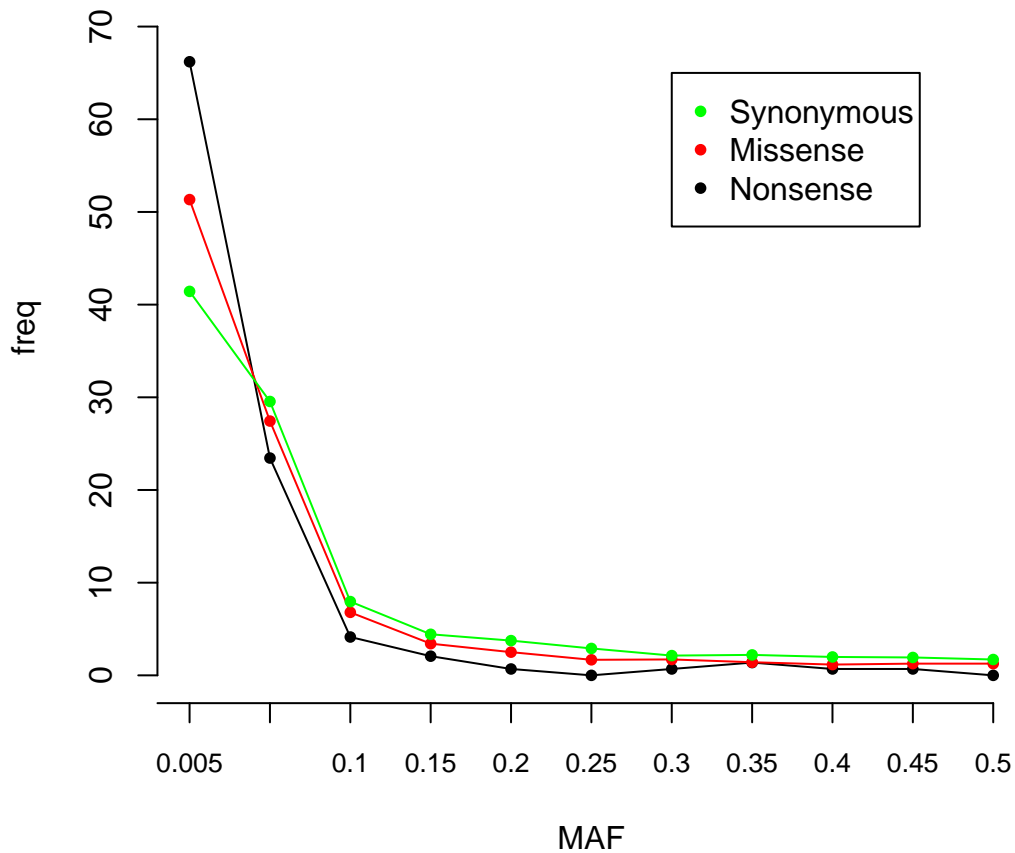

Supplement: Figure S3 — Site frequency spectrum for the synonymous, missense and nonsense variants in the French-Canadian population. (PDF) [file pgen.1003815.s003.pdf]

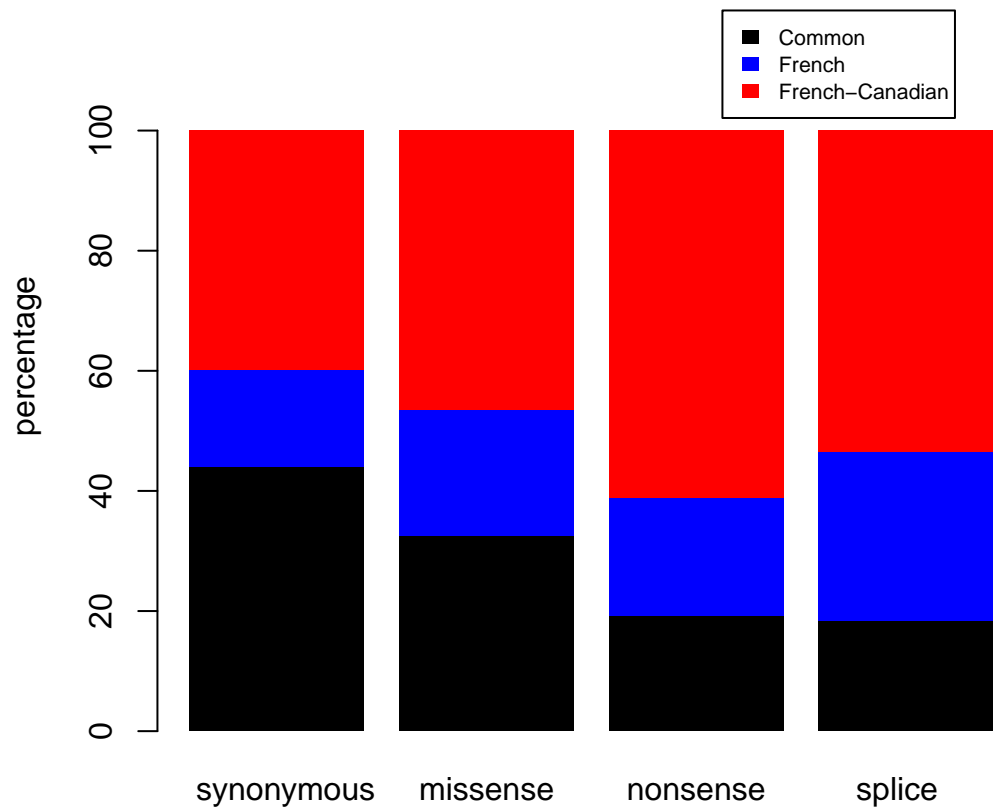

Supplement: Figure S4 — Percentage of shared and private variants between the French (N = 30) and the French-Canadian populations (N = 109) in this study. (PDF) [file pgen.1003815.s004.pdf]

A

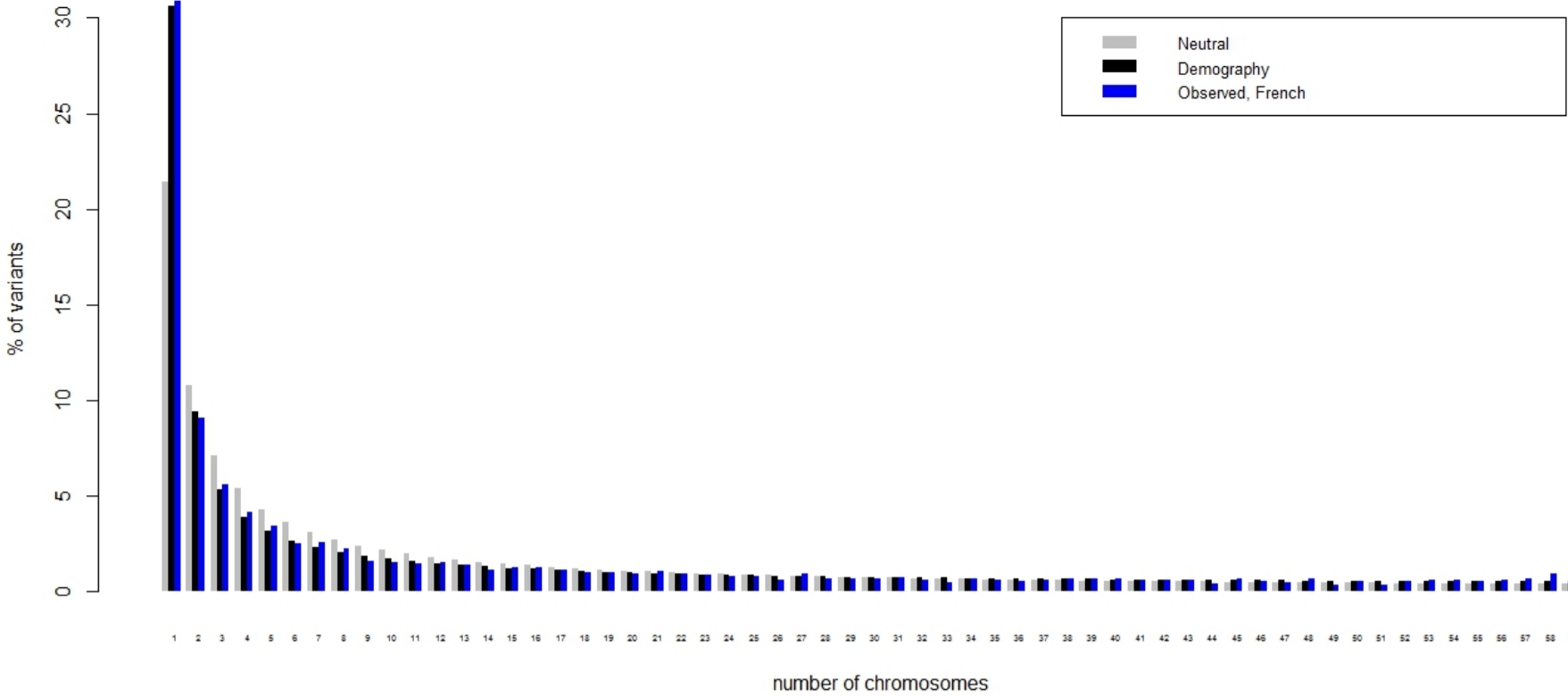

B

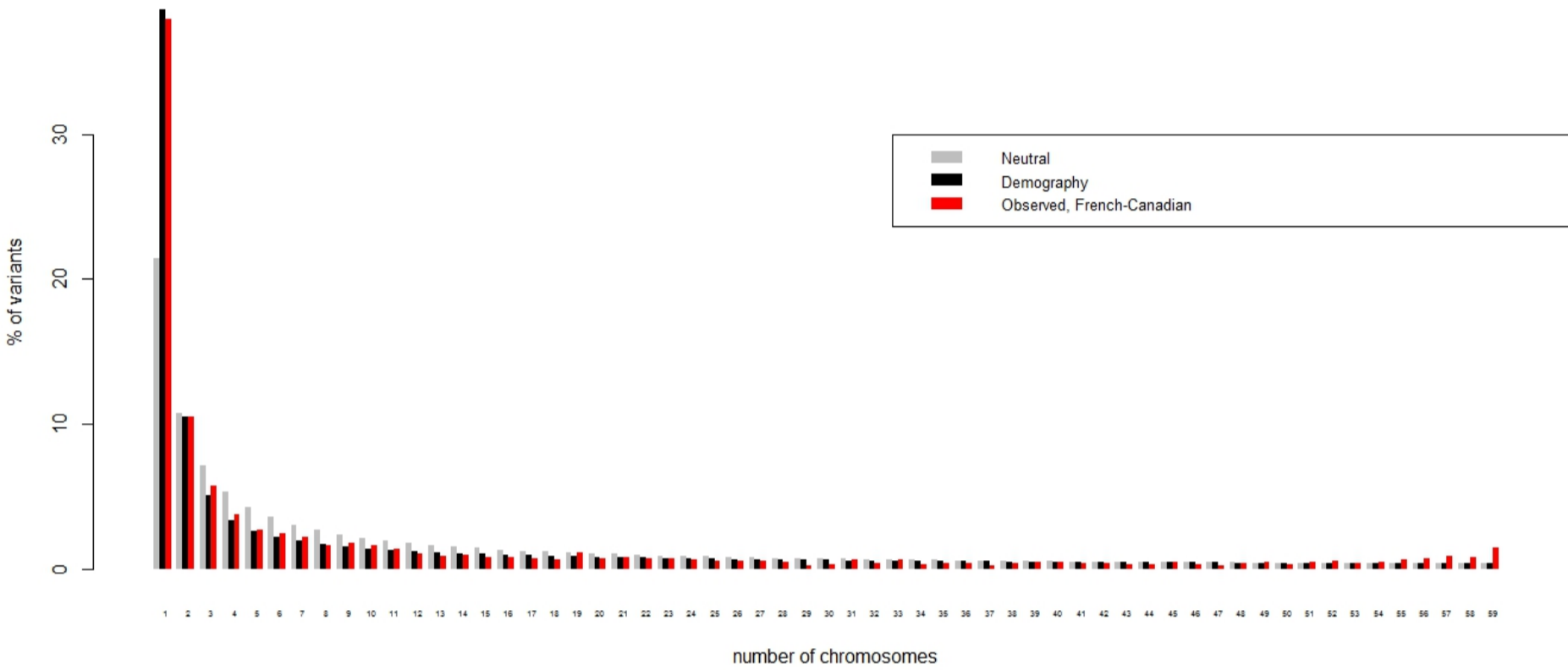

C

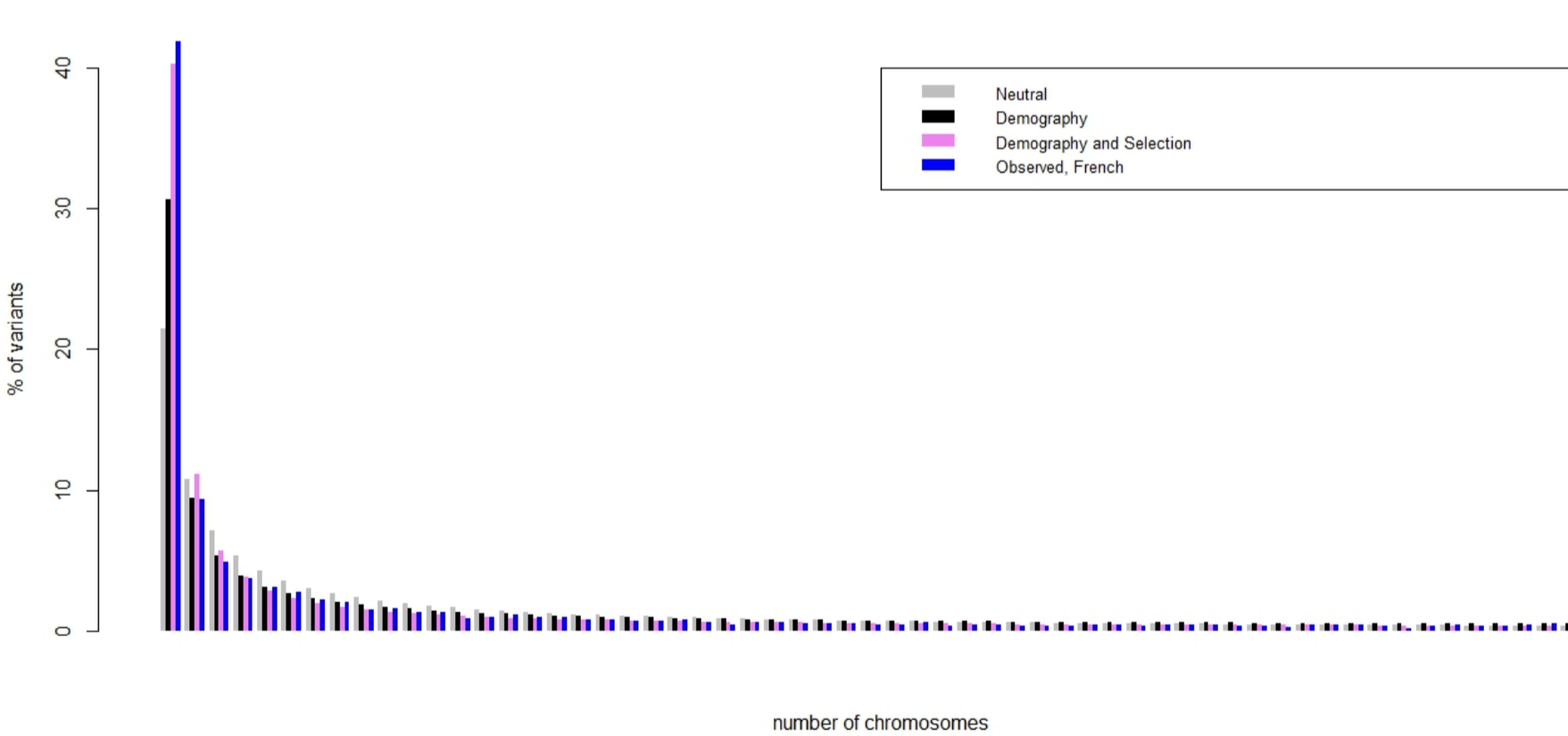

D

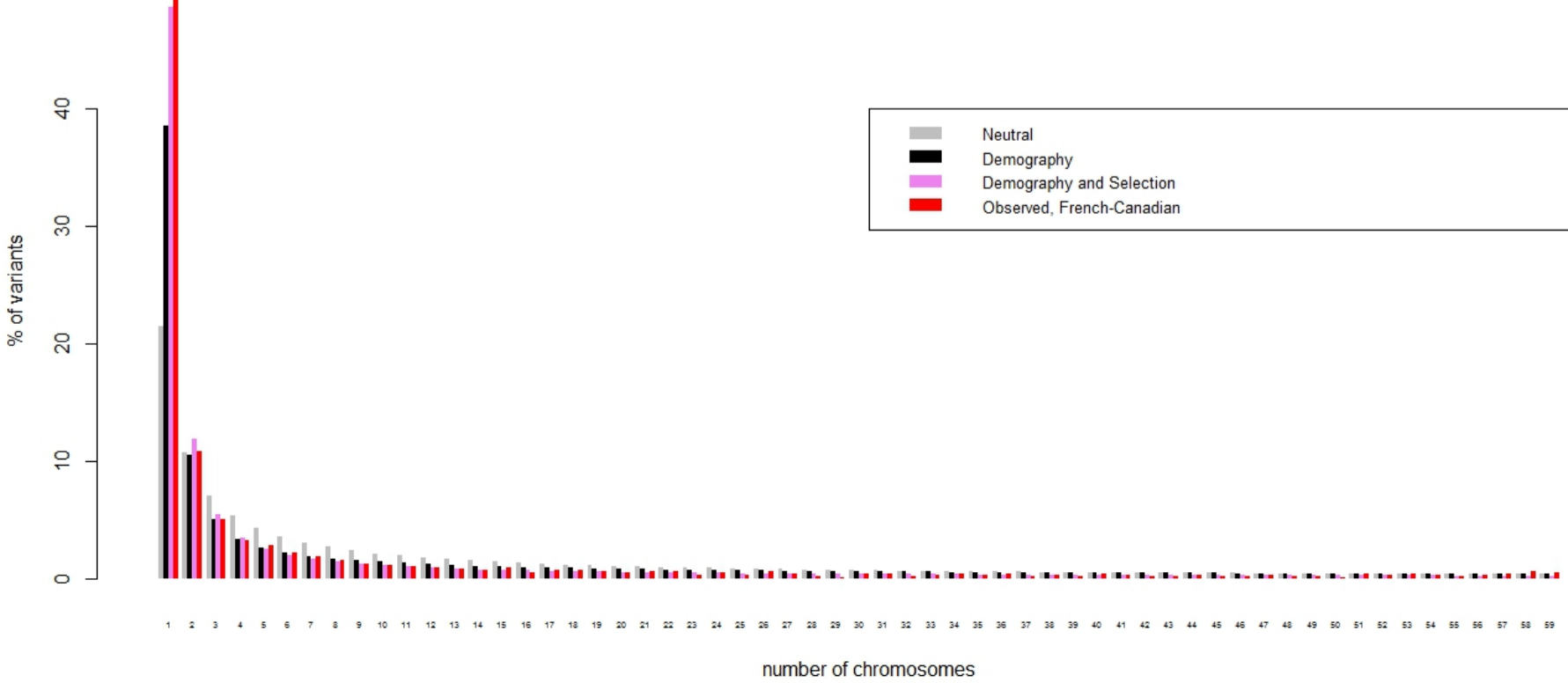

Supplement: Figure S5 — Observed and expected folded site frequency spectrum using prfreq. Observed and expected site frequency distributions for the synonymous SNPs in the French population (A), synonymous SNPs in the French-Canadian population (B), nonsynonymous SNPs in the French Population (C), and nonsynonymous SNPs in the French-Canadian population (D). Expected distributions have been obtained with a neutral model not including demography, a model with demography, and for the nonsynonymous variants a model with demography and selection is also included (see parameters in Table S3). (PDF) [file pgen.1003815.s005.pdf]

A

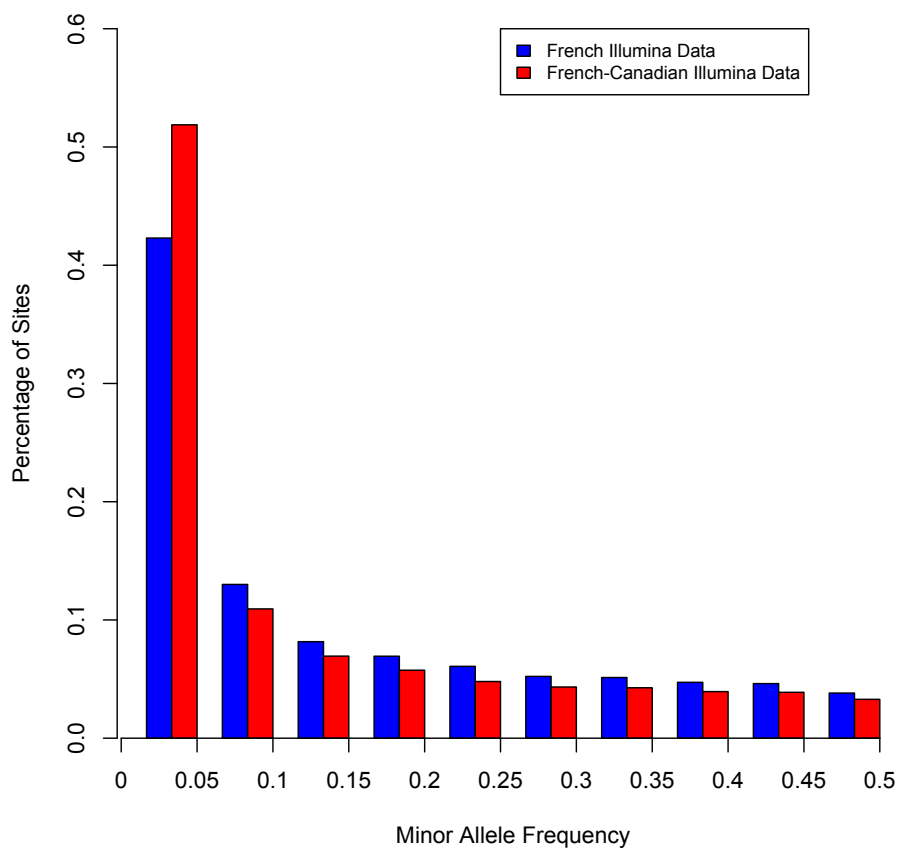

B

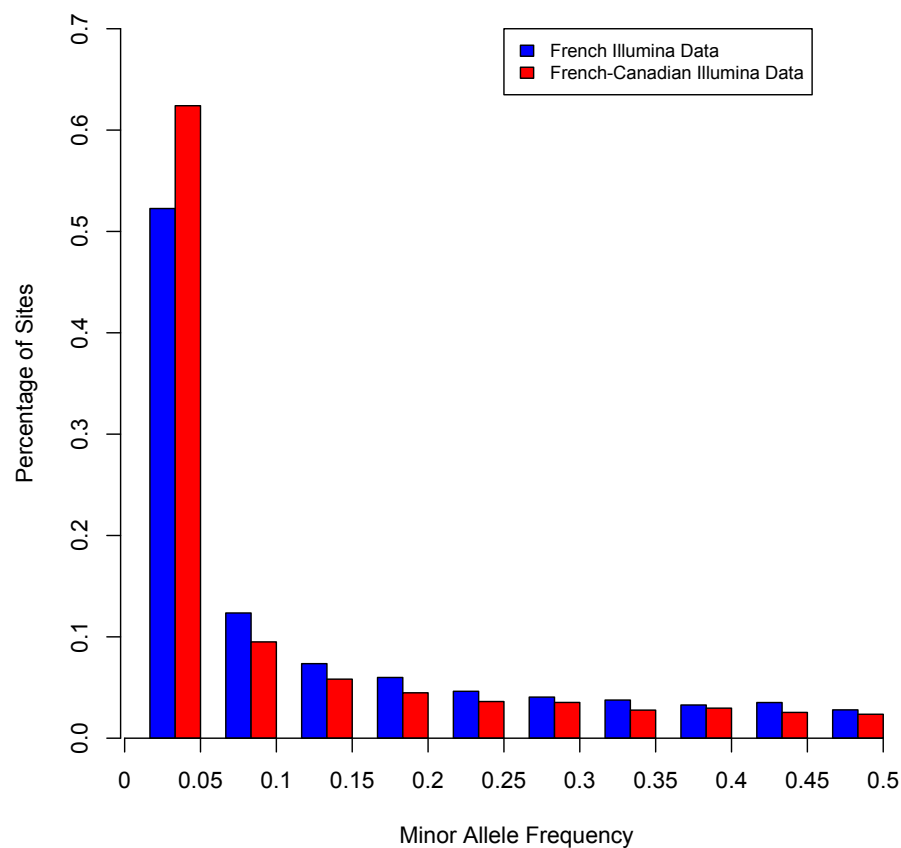

C

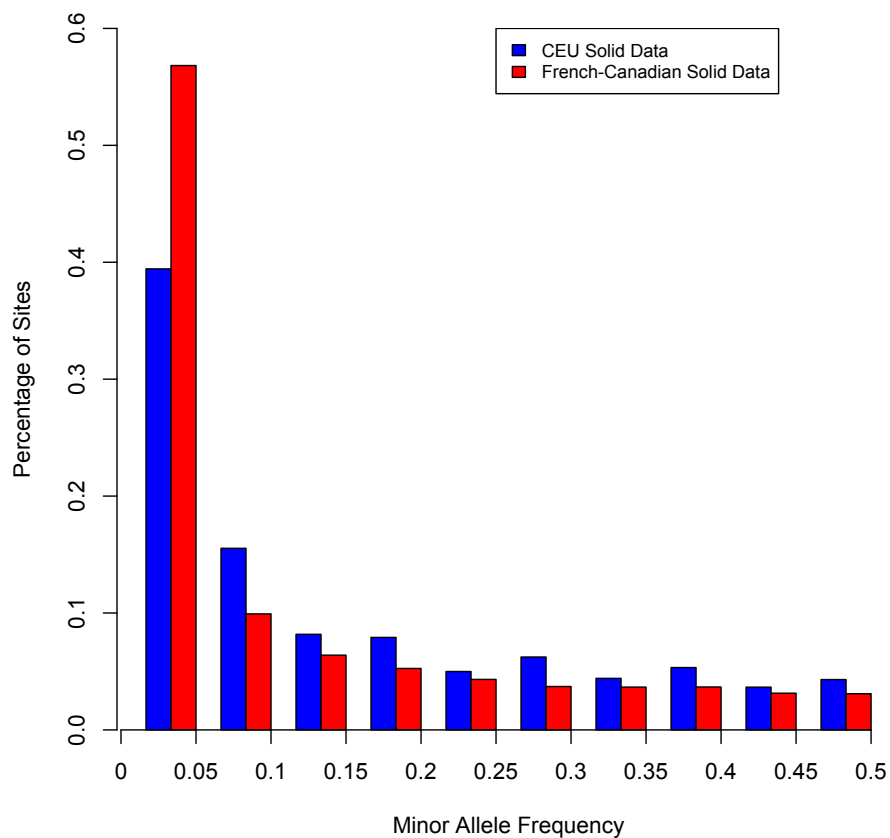

D

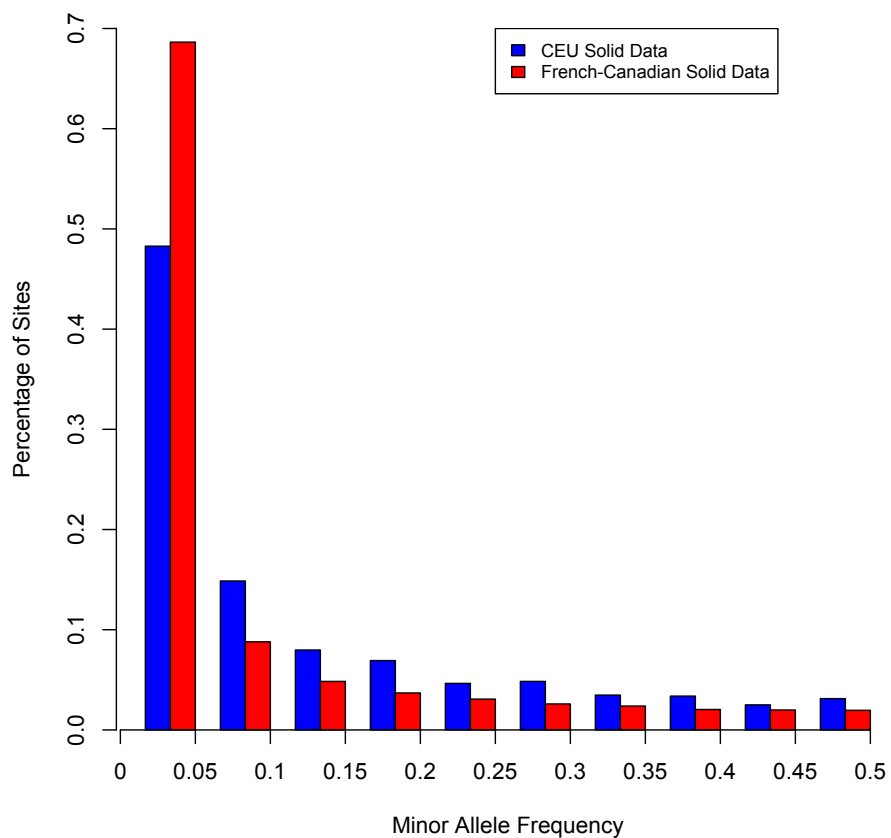

Supplement: Figure S6 — A) Site frequency spectrum in the French and the French-Canadian populations using Illumina datasets for the synonymous variants; B) Site frequency spectrum in the French and the French-Canadian populations using Illumina datasets for the missense variants; C) Site frequency spectrum in the CEU and the French-Canadian populations using SOLiD datasets for the synonymous variants; D) Site frequency spectrum in the CEU and the French-Canadian populations using SOLiD datasets for the missense variants. (PDF) [file pgen.1003815.s006.pdf]

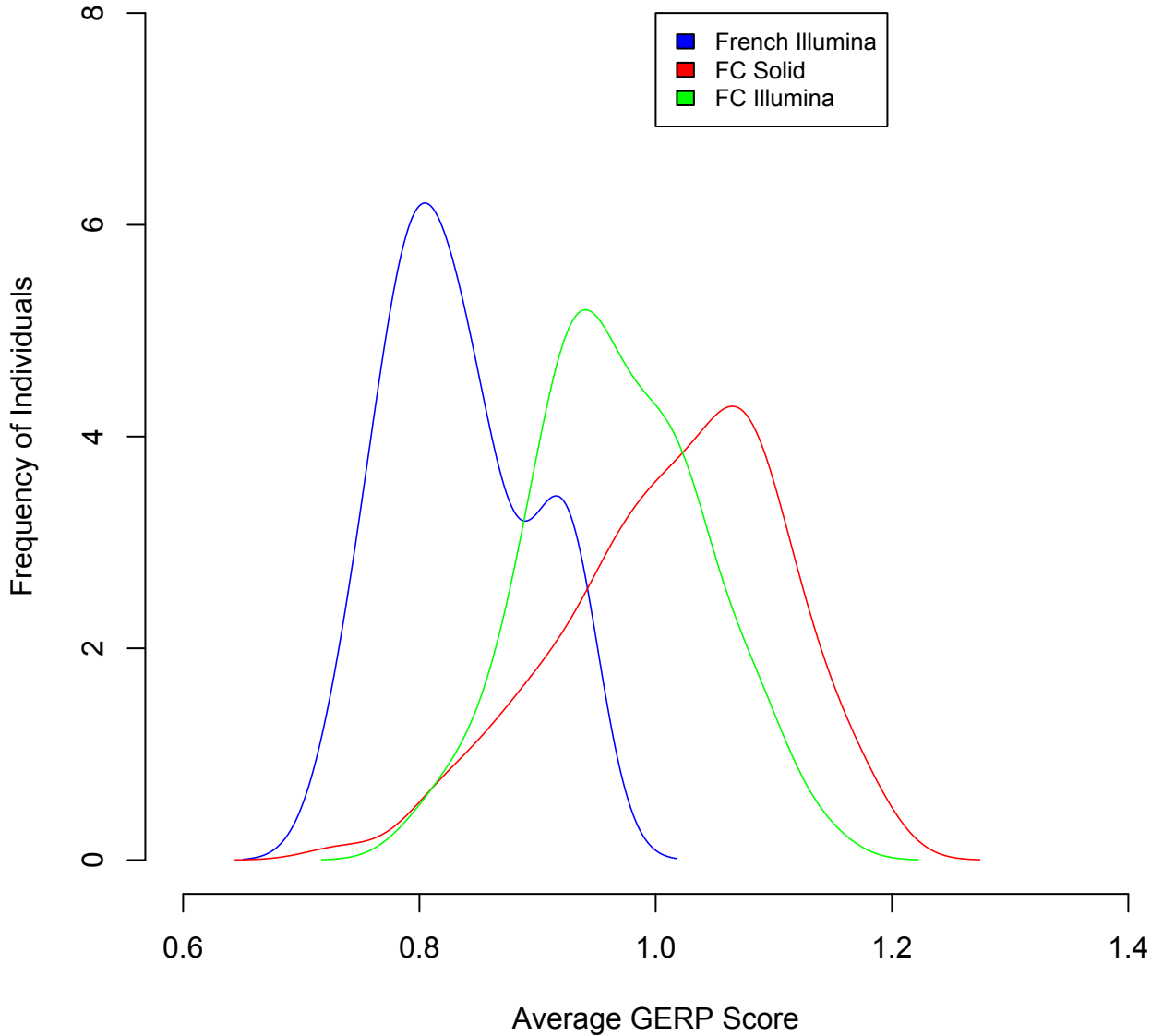

Supplement: Figure S7 — Distributions of the average GERP scores at functional sites per individual in the French and the French-Canadian populations. GERP scores are averaged per individual by using only sites at which each individual carries the minor allele. (PDF) [file pgen.1003815.s007.pdf]

## Nucleotidic changes

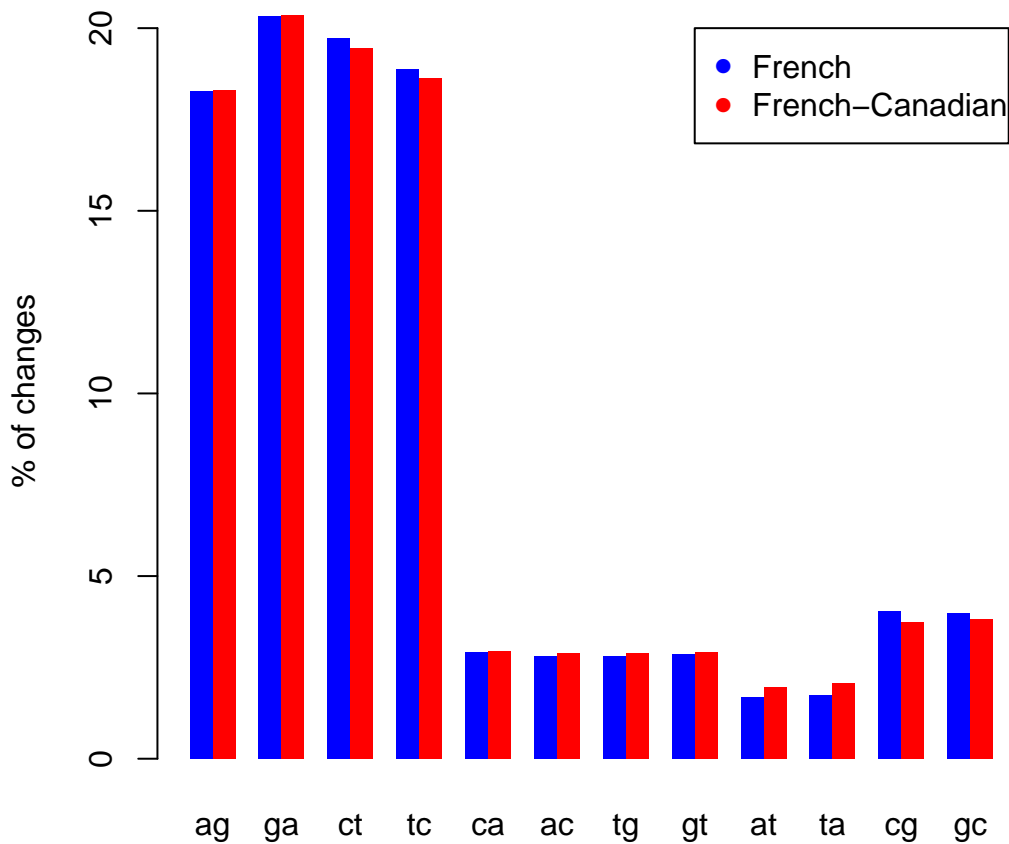

Supplement: Figure S8 — The proportions of the twelve possible nucleotide changes in each population. (PDF) [file pgen.1003815.s008.pdf]

A

## French Canadian

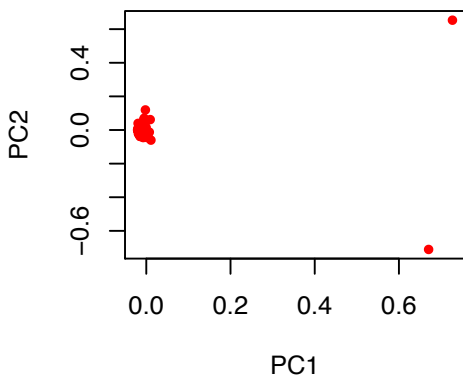

B

## French Canadian

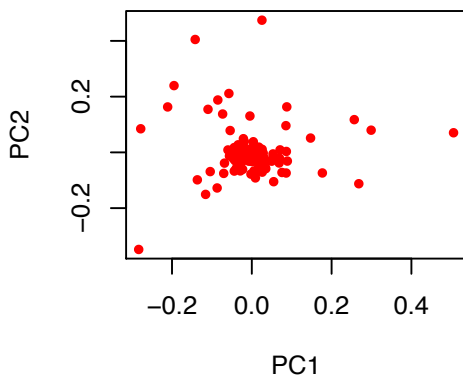

C

## French

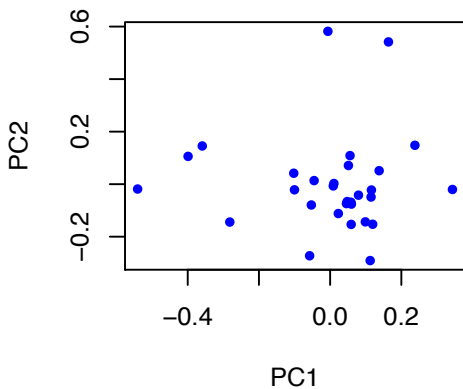

D

## French and French Canadian

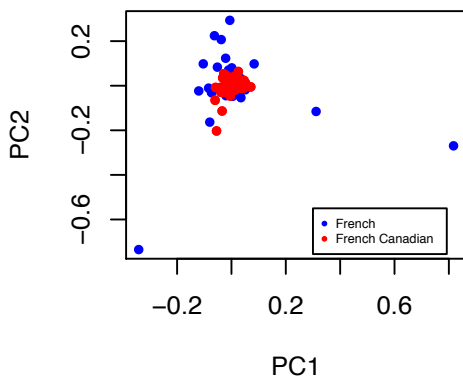

Supplement: Figure S9 — Principal Component Analysis with A) the 114 French-Canadian samples; B) the 109 French-Canadian samples after removing five genetic outliers; C) the 30 French samples (no genetic outliers were detected); D) the 109 French-Canadian and the 30 French samples. (PDF) [file pgen.1003815.s009.pdf]

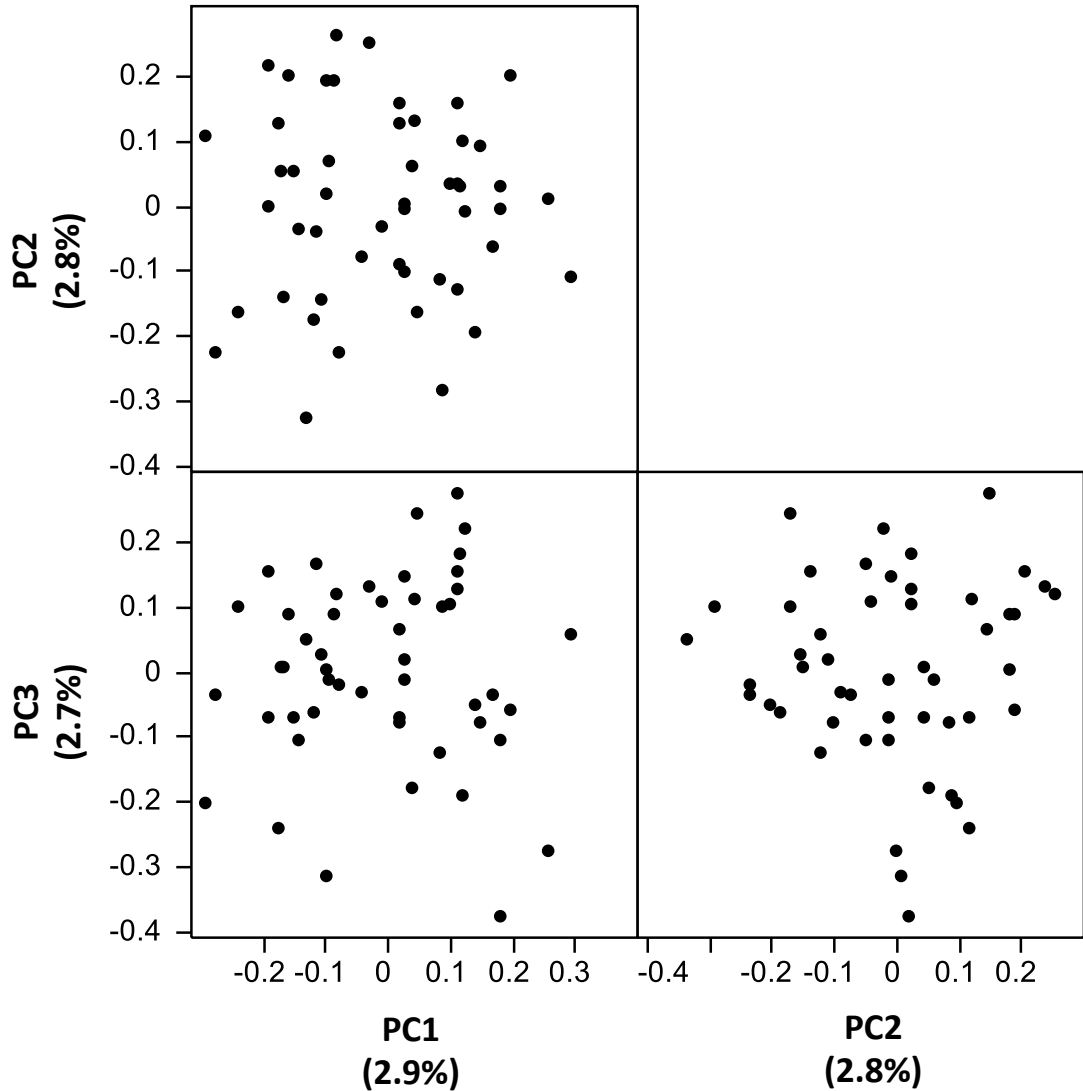

Supplement: Figure S10 — Principal Component Analysis of the 50 French Canadians sequenced using the Illumina technology. (PDF) [file pgen.1003815.s010.pdf]
