# Supplementary material for: Whole-Exome Sequencing Reveals a Rapid Change in the Frequency of Rare Functional Variants in a Founding Population of Humans
Source: PLoS Genet. 2013 Sep 26;9(9):e1003815. doi: 10.1371/journal.pgen.1003815 (PMC3784517; doi:10.1371/journal.pgen.1003815)
Supplement: Table S1 — Total number of SNPs in the 38 Mb targeted regions in the French and the French-Canadian populations. (DOCX) [file pgen.1003815.s011.docx]

|  | Total SNPs | Nonsense | Missense | Synonymous | Splice | Non-Func |
| --- | --- | --- | --- | --- | --- | --- |
| French | 46,662 | 204 | 16,219 | 15,764 | 86 | 14,389 |
| French-Canadian | 64,631 | 422 | 23,996 | 20,067 | 133 | 20,013 |
