# Supplementary material for: Whole-Exome Sequencing Reveals a Rapid Change in the Frequency of Rare Functional Variants in a Founding Population of Humans
Source: PLoS Genet. 2013 Sep 26;9(9):e1003815. doi: 10.1371/journal.pgen.1003815 (PMC3784517; doi:10.1371/journal.pgen.1003815)
Supplement: Table S2 — prfreq maximum likelihood estimates of neutral, demographic and selective models for the French Population. (DOCX) [file pgen.1003815.s012.docx]

| **Scenario** | **Mutations^a^** | **Model** | **Log Likelihood** | **Fixed parameters** | **Estimated parameters** | ***P*^b^** | **Goodness of fit^c^** |
| --- | --- | --- | --- | --- | --- | --- | --- |
| FR1 | Silent | Stationary | 49694.06 | - | - | - | p<2.2e-16 |
| FR2 | Silent | Demography (contraction/expansion) | 49898.92 | - | TAU = 0.0008  OMEGA = 0.04 | < 0.001  (FR2 vs FR1) |  |
| FR3 | Silent | Demography (bottleneck) | 49990.87 | - | TAU = 0.41  OMEGA = 0.79  TAU_B = 0.399  OMEGA_B = 0.18 | < 0.001  (FR3 vs FR2) | p=0.9082 |
| FR4 | Silent | Demography (bottleneck)  + Selection | 49991.07 | TAU = 0.41  OMEGA = 0.79  TAU_B = 0.399  OMEGA_B = 0.18 | P = 0.95  γ = - 67 | 0.82  (FR4 vs FR3) |  |
| FR5 | Missense | Demography (bottleneck) | 57046.22 | TAU = 0.41  OMEGA = 0.79  TAU_B = 0.399  OMEGA_B = 0.18 | - | - | p<2.2e-16 |
| FR6 | Missense | Demography (bottleneck)  + Selection | 57324.93 | TAU = 0.41  OMEGA = 0.79  TAU_B = 0.399  OMEGA_B = 0.18 | P = 0.46  γ = - 115 | < 0.001  (FR6 vs FR5) | p=0.6581 |
| FR7 | Missense | Demography (bottleneck)  + Selection | 57320.73 | TAU = 0.41  OMEGA = 0.79  TAU_B = 0.399  OMEGA_B = 0.18  P = 0.46  γ = - 82 (estimated in French-Canadian) | - | < 0.001  (FR6 vs FR7) |  |

^a^Autosomal mutations only. ^b^χ^2^ (*P* value) with degrees of freedom being the difference of the number of estimated parameters. TAU is the time in generations since the non-stationary dynamics, scaled by 2*Ncurr. TAU B is the scaled time of the bottleneck. OMEGA is the ratio of ancestral to current Ne. OMEGA B is the ratio of bottleneck to current Ne. P is the probability of a mutation of being neutral. γ is the population selection parameter (γ = *Ne(s)*). ^c^ Goodness of fit test compared to observed data using Kolmogorov-Smirnov tests.
