# Supplementary material for: Whole-Exome Sequencing Reveals a Rapid Change in the Frequency of Rare Functional Variants in a Founding Population of Humans
Source: PLoS Genet. 2013 Sep 26;9(9):e1003815. doi: 10.1371/journal.pgen.1003815 (PMC3784517; doi:10.1371/journal.pgen.1003815)
Supplement: Table S3 — prfreq maximum likelihood estimates of neutral, demographic and selective models for the French Population. (DOCX) [file pgen.1003815.s013.docx]

| **Scenario** | **Mutations^a^** | **Model** | **Log Likelihood** | **Fixed parameters** | **Estimated parameters** | ***P*^b^** | **Goodness of fit^c^** |
| --- | --- | --- | --- | --- | --- | --- | --- |
| FC1 | Silent | Stationary | 25076.11 | - | - | - | p<2.2e-16 |
| FC2 | Silent | Demography (contraction/expansion) | 25306.09 | - | TAU = 0.1  OMEGA = 0.406 | < 0.001  (FC2 vs FC1) |  |
| FC3 | Silent | Demography (bottleneck) | 25444.90 | - | TAU = 0.132  OMEGA = 0.26  TAU_B = 0.11536  OMEGA_B = 0.125 | < 0.001  (FC3 vs FC2) | p=0.3261 |
| FC4 | Silent | Demography (bottleneck)  + Selection | 25445.04 | TAU = 0.132  OMEGA = 0.26  TAU_B = 0.11536  OMEGA_B = 0.125 | P = 0.95  γ = - 50 | 0.87  (FC4 vs FC3) |  |
| FC5 | Missense | Demography (bottleneck) | 34932.82 | TAU = 0.132  OMEGA = 0.26  TAU_B = 0.11536  OMEGA_B = 0.125 | - | - | p<2.2e-16 |
| FC6 | Missense | Demography (bottleneck)  + Selection | 35131.65 | TAU = 0.132  OMEGA = 0.26  TAU_B = 0.11536  OMEGA_B = 0.125 | P = 0.4  γ = -82 | < 0.001  (FC6 vs FC5) | p=0.9596 |
| FC7 | Missense | Demography (bottleneck)  + Selection | 35125.79 | TAU = 0.132  OMEGA = 0.26  TAU_B = 0.11536  OMEGA_B = 0.125  P = 0.4  γ = - 115 (estimated in French) | - | < 0.001  (FC6 vs FC7) |  |

^a^Autosomal mutations only. ^b^χ^2^ (*P* value) with degrees of freedom being the difference of the number of estimated parameters. TAU is the time in generations since the non-stationary dynamics, scaled by 2*Ncurr. TAU B is the scaled time of the bottleneck. OMEGA is the ratio of ancestral to current Ne. OMEGA B is the ratio of bottleneck to current Ne. P is the probability of a mutation of being neutral. γ is the population selection parameter (γ = *Ne(s)*). ^c^ Goodness of fit test compared to observed data using Kolmogorov-Smirnov tests.
