# Supplementary material for: Whole-Exome Sequencing Reveals a Rapid Change in the Frequency of Rare Functional Variants in a Founding Population of Humans
Source: PLoS Genet. 2013 Sep 26;9(9):e1003815. doi: 10.1371/journal.pgen.1003815 (PMC3784517; doi:10.1371/journal.pgen.1003815)
Supplement: Table S4 — Summary values for tests for rare and damaging variants in the French and French-Canadian populations. Rare variants are defined as those with a minor allele frequency less that 5%. (DOCX) [file pgen.1003815.s014.docx]

| **Test** | **French Illumina Dataset** | **CEU Solid Dataset** | **French Canadian Illumina Dataset** | | **French Canadian Solid Dataset** | |
| --- | --- | --- | --- | --- | --- | --- |
|  | **Value** |  | **Value** | **p-value vs French Illumina dataset** | **Value** | **p-value vs French Illumina dataset** |
| **Variants with MAF ≤ 5%** | **45.75%** | **44.16%** | **57.39%** | **<0.01** | **60.80%** | **<0.01** |
| **Missense:Synonymous ratio for rare variants** | **1.31** | **1.33** | **1.39** | **<0.01** | **1.47** | **<0.01** |
| **Proportion of missense sites that are damaging with polyphen** | **45.5%** | **47.0%** | **48.2%** | **<0.01** | **49.5%** | **<0.01** |
| **Average GERP score for rare missense variants** | **2.067** | **2.101** | **2.194** | **<0.01** | **2.174** | **<0.01** |
