# Supplementary material for: Whole-Exome Sequencing Reveals a Rapid Change in the Frequency of Rare Functional Variants in a Founding Population of Humans
Source: PLoS Genet. 2013 Sep 26;9(9):e1003815. doi: 10.1371/journal.pgen.1003815 (PMC3784517; doi:10.1371/journal.pgen.1003815)
Supplement: Table S5 — Sequencing statistics for the 144 samples included in this study. (DOCX) [file pgen.1003815.s015.docx]

| ID | Cohort^*^ | Pop^†^ | Total  Bases | Total  Positions | Genome  Coverage | Mean  Coverage | Mean  Base  quality | Mean  Mapping  quality | RMS  Mapping  quality | Variants | SNPs  Q20 | Hetero | Hom |
| --- | --- | --- | --- | --- | --- | --- | --- | --- | --- | --- | --- | --- | --- |
| 375_F | ALL | FC | 354339626 | 29758698 | 0.79 | 11.91 | 23.25 | 33.09 | 40.05 | 12162 | 9055 | 4300 | 4755 |
| 375_M | ALL | FC | 355065848 | 29691507 | 0.79 | 11.96 | 22.88 | 32.72 | 39.71 | 12010 | 8970 | 4194 | 4776 |
| 380_F | ALL | FC | 375033466 | 29650017 | 0.78 | 12.65 | 23.03 | 33.30 | 40.14 | 12235 | 9369 | 4421 | 4948 |
| 380_M | ALL | FC | 436987305 | 30066772 | 0.80 | 14.53 | 22.91 | 33.55 | 40.12 | 12971 | 10046 | 4904 | 5142 |
| 390_F | ALL | FC | 487011622 | 30282533 | 0.80 | 16.08 | 23.64 | 33.87 | 40.16 | 13353 | 10580 | 5432 | 5148 |
| 390_M | ALL | FC | 383439630 | 29630038 | 0.78 | 12.94 | 24.19 | 33.29 | 40.16 | 12619 | 9430 | 4299 | 5131 |
| 392_F | ALL | FC | 500748003 | 31919022 | 0.84 | 15.69 | 23.48 | 32.52 | 38.64 | 14259 | 10984 | 5257 | 5727 |
| 392_M | ALL | FC | 441072923 | 31449788 | 0.83 | 14.02 | 22.94 | 32.20 | 38.45 | 13968 | 10745 | 5198 | 5547 |
| 399_F | ALL | FC | 186426639 | 28341857 | 0.75 | 6.58 | 22.52 | 30.65 | 39.46 | 9874 | 6209 | 2430 | 3779 |
| 399_M | ALL | FC | 298752599 | 30267940 | 0.80 | 9.87 | 22.66 | 32.18 | 39.70 | 11846 | 8450 | 3632 | 4818 |
| 420_F | ALL | FC | 459657291 | 31815630 | 0.84 | 14.45 | 23.92 | 33.68 | 40.11 | 14326 | 11127 | 5273 | 5854 |
| 420_M | ALL | FC | 374353999 | 31106360 | 0.82 | 12.03 | 23.65 | 31.97 | 38.85 | 12994 | 9735 | 4574 | 5161 |
| 424_F | ALL | FC | 416731079 | 31820250 | 0.84 | 13.10 | 23.37 | 32.18 | 38.60 | 13946 | 10563 | 5225 | 5338 |
| 424_M | ALL | FC | 459521410 | 31139228 | 0.82 | 14.76 | 23.03 | 31.51 | 37.73 | 13343 | 10181 | 4894 | 5287 |
| 443_F | ALL | FC | 426566291 | 31701235 | 0.84 | 13.46 | 23.63 | 33.35 | 39.96 | 13877 | 10665 | 5135 | 5530 |
| 443_M | ALL | FC | 479304177 | 31799292 | 0.84 | 15.07 | 23.70 | 33.76 | 40.06 | 14355 | 11148 | 5527 | 5621 |
| 579_F | ALL | FC | 411318741 | 31664153 | 0.84 | 12.99 | 23.51 | 33.20 | 39.85 | 13666 | 10374 | 4778 | 5596 |
| 579_M | ALL | FC | 277417203 | 30002621 | 0.79 | 9.25 | 22.54 | 31.99 | 39.66 | 11492 | 8219 | 3598 | 4621 |
| 580_F | ALL | FC | 336418939 | 31007128 | 0.82 | 10.85 | 22.96 | 32.80 | 40.00 | 12716 | 9312 | 4220 | 5092 |
| 580_M | ALL | FC | 387882379 | 31173498 | 0.82 | 12.44 | 23.35 | 33.04 | 39.76 | 13330 | 10106 | 4477 | 5629 |
| 595_F | ALL | FC | 510069769 | 32303598 | 0.85 | 15.79 | 24.14 | 33.70 | 39.81 | 14880 | 11720 | 5756 | 5964 |
| 595_M | ALL | FC | 253399078 | 29614267 | 0.78 | 8.56 | 22.51 | 31.97 | 40.06 | 11350 | 7882 | 3298 | 4584 |
| 614_F | ALL | FC | 401854810 | 30465958 | 0.81 | 13.19 | 22.87 | 31.74 | 38.27 | 12599 | 9611 | 4532 | 5079 |
| 614_M | ALL | FC | 312227082 | 30022950 | 0.79 | 10.40 | 22.16 | 30.60 | 37.72 | 11317 | 8225 | 3590 | 4635 |
| 617_F | ALL | FC | 246935738 | 29188828 | 0.77 | 8.46 | 21.86 | 30.21 | 37.91 | 10751 | 7469 | 3011 | 4458 |
| 617_M | ALL | FC | 168015971 | 27404454 | 0.72 | 6.13 | 21.04 | 28.83 | 37.53 | 8786 | 5492 | 2056 | 3436 |
| 657_F | ALL | FC | 237397649 | 28969697 | 0.77 | 8.19 | 21.92 | 29.62 | 37.42 | 10198 | 6965 | 2761 | 4204 |
| 657_M | ALL | FC | 223068344 | 28777834 | 0.76 | 7.75 | 21.80 | 29.15 | 37.05 | 9871 | 6646 | 2635 | 4011 |
| 685_F | ALL | FC | 433567210 | 30942978 | 0.82 | 14.01 | 22.88 | 31.41 | 37.81 | 12945 | 9813 | 4593 | 5220 |
| 685_M | ALL | FC | 432756403 | 30688391 | 0.81 | 14.10 | 23.13 | 31.88 | 38.27 | 13005 | 9983 | 4876 | 5107 |
| 728_F | ALL | FC | 428963599 | 29789845 | 0.79 | 14.40 | 23.27 | 33.59 | 40.14 | 12914 | 10029 | 4986 | 5043 |
| 728_M | ALL | FC | 408475540 | 30357905 | 0.80 | 13.46 | 23.26 | 33.41 | 40.13 | 12771 | 9823 | 4178 | 5645 |
| 752_F | ALL | FC | 358446607 | 29337337 | 0.78 | 12.22 | 22.86 | 33.22 | 40.24 | 12303 | 9282 | 4440 | 4842 |
| 752_M | ALL | FC | 425703452 | 29779147 | 0.79 | 14.30 | 22.95 | 33.48 | 40.01 | 12799 | 9884 | 4829 | 5055 |
| 761_F | ALL | FC | 480273396 | 32078699 | 0.85 | 14.97 | 23.35 | 32.30 | 38.54 | 14107 | 10838 | 5010 | 5828 |
| 761_M | ALL | FC | 492011188 | 32272083 | 0.85 | 15.25 | 23.58 | 32.66 | 38.82 | 14320 | 10981 | 5388 | 5593 |
| 762_F | ALL | FC | 412920137 | 30271974 | 0.80 | 13.64 | 23.07 | 31.75 | 38.29 | 12596 | 9510 | 4441 | 5069 |
| 762_M | ALL | FC | 405443404 | 30596507 | 0.81 | 13.25 | 22.87 | 31.52 | 38.05 | 12738 | 9603 | 4586 | 5017 |
| 764_F | ALL | FC | 354521240 | 29218738 | 0.77 | 12.13 | 23.12 | 33.09 | 40.09 | 12022 | 9052 | 4260 | 4792 |
| 764_M | ALL | FC | 409143255 | 29854241 | 0.79 | 13.70 | 23.09 | 33.40 | 40.03 | 12651 | 9818 | 4694 | 5124 |
| 767_F | ALL | FC | 480292810 | 31531949 | 0.83 | 15.23 | 23.47 | 32.17 | 38.28 | 13732 | 10574 | 5602 | 4972 |
| 767_M | ALL | FC | 534300583 | 31428420 | 0.83 | 17.00 | 23.49 | 32.38 | 38.31 | 14040 | 10942 | 5485 | 5457 |
| 777_F | ALL | FC | 371802185 | 30252742 | 0.80 | 12.29 | 22.71 | 30.48 | 37.09 | 12167 | 8946 | 4080 | 4866 |
| 777_M | ALL | FC | 510038711 | 31227646 | 0.83 | 16.33 | 23.44 | 32.50 | 38.40 | 13927 | 10995 | 5427 | 5568 |
| 790_F | ALL | FC | 401339951 | 29959796 | 0.79 | 13.40 | 23.32 | 32.97 | 39.66 | 12835 | 9822 | 4720 | 5102 |
| 790_M | ALL | FC | 466044304 | 30170435 | 0.80 | 15.45 | 23.58 | 33.66 | 40.03 | 13219 | 10387 | 5218 | 5169 |
| 794_F | ALL | FC | 331862108 | 29463279 | 0.78 | 11.26 | 22.88 | 32.78 | 40.02 | 12006 | 8839 | 3999 | 4840 |
| 794_M | ALL | FC | 329673885 | 29350179 | 0.78 | 11.23 | 23.02 | 32.71 | 39.97 | 11441 | 8461 | 3860 | 4601 |
| ID_11M | PID | FC | 744253947 | 36405473 | 0.96 | 20.44 | 25.33 | 72.78 | 55.61 | 22144 | 18548 | 10321 | 8227 |
| ID_11P | PID | FC | 701826802 | 36272800 | 0.96 | 19.35 | 24.52 | 69.28 | 54.74 | 22904 | 17442 | 9875 | 7567 |
| ID_12M | PID | FC | 830753864 | 36532666 | 0.97 | 22.74 | 25.97 | 73.71 | 55.68 | 23458 | 19540 | 11171 | 8369 |
| ID_13P | PID | FC | 792855783 | 36405710 | 0.96 | 21.78 | 24.52 | 68.74 | 54.29 | 21676 | 17475 | 9627 | 7848 |
| ID_15M | PID | FC | 1010504089 | 34967682 | 0.92 | 28.90 | 24.97 | 74.11 | 55.40 | 20472 | 17864 | 10438 | 7426 |
| ID_15P | PID | FC | 1049821908 | 35107459 | 0.93 | 29.90 | 25.16 | 73.85 | 55.34 | 20608 | 18069 | 10370 | 7699 |
| ID_2P | PID | FC | 868554171 | 34977846 | 0.93 | 24.83 | 24.85 | 72.91 | 55.23 | 20157 | 17262 | 9777 | 7485 |
| ID_3M | PID | FC | 1144320276 | 36607509 | 0.97 | 31.26 | 25.55 | 74.00 | 55.49 | 23770 | 20164 | 11796 | 8368 |
| ID_3P | PID | FC | 1037555949 | 36585214 | 0.97 | 28.36 | 25.98 | 73.57 | 55.46 | 24722 | 20069 | 11673 | 8396 |
| ID_5M | PID | FC | 970901072 | 36664005 | 0.97 | 26.48 | 25.37 | 71.94 | 55.21 | 23708 | 19278 | 11171 | 8107 |
| ID_6M | PID | FC | 887756852 | 36580909 | 0.97 | 24.27 | 25.75 | 72.97 | 55.46 | 23236 | 19096 | 11040 | 8056 |
| ID_7M | PID | FC | 794725999 | 34512626 | 0.91 | 23.03 | 24.39 | 72.44 | 55.18 | 19314 | 16447 | 9426 | 7021 |
| ID_7P | PID | FC | 975910482 | 35124870 | 0.93 | 27.78 | 24.76 | 73.16 | 55.25 | 20540 | 17738 | 9932 | 7806 |
| ID_8M | PID | FC | 2498642411 | 36694255 | 0.97 | 68.09 | 26.92 | 37.38 | 40.62 | 23846 | 21220 | 12410 | 8810 |
| ID_9M | PID | FC | 637414775 | 34661643 | 0.92 | 18.39 | 23.99 | 72.10 | 55.17 | 19610 | 16316 | 9081 | 7235 |
| ID_9P | PID | FC | 968347801 | 36795545 | 0.97 | 26.32 | 26.22 | 74.83 | 55.86 | 24091 | 20297 | 11793 | 8504 |
| S00302 | ASD | FC | 645278236 | 35374994 | 0.94 | 18.24 | 27.12 | 73.59 | 55.80 | 21236 | 17497 | 9672 | 7825 |
| S00303 | ASD | FC | 643145354 | 35057166 | 0.93 | 18.35 | 26.01 | 71.80 | 55.39 | 22705 | 18784 | 11217 | 7567 |
| S00314 | ASD | FC | 680318327 | 35103953 | 0.93 | 19.38 | 27.32 | 75.28 | 56.20 | 20887 | 17492 | 9643 | 7849 |
| S00332 | ASD | FC | 592875060 | 33312364 | 0.88 | 17.80 | 25.06 | 69.31 | 54.61 | 17403 | 13754 | 7564 | 6190 |
| S00353 | ASD | FC | 596043789 | 33350112 | 0.88 | 17.87 | 25.24 | 69.72 | 54.77 | 17788 | 14106 | 7838 | 6268 |
| S00379 | ASD | FC | 564639868 | 35251165 | 0.93 | 16.02 | 27.51 | 74.12 | 56.11 | 20178 | 16657 | 9085 | 7572 |
| S00380 | ASD | FC | 549287818 | 33747580 | 0.89 | 16.28 | 26.17 | 70.25 | 54.99 | 17610 | 14059 | 7724 | 6335 |
| S00397 | ASD | FC | 556515276 | 33660785 | 0.89 | 16.53 | 26.28 | 70.00 | 54.92 | 17121 | 13786 | 7256 | 6530 |
| S00398 | ASD | FC | 659447996 | 35005921 | 0.93 | 18.84 | 26.35 | 72.71 | 55.43 | 23273 | 19726 | 11733 | 7993 |
| S00427 | ASD | FC | 610882953 | 33404703 | 0.88 | 18.29 | 25.02 | 69.56 | 54.63 | 17698 | 14062 | 7654 | 6408 |
| S00443 | ASD | FC | 433975947 | 32603081 | 0.86 | 13.31 | 23.14 | 66.80 | 54.28 | 16041 | 12110 | 6416 | 5694 |
| S00448 | ASD | FC | 586718417 | 34303812 | 0.91 | 17.10 | 25.90 | 71.50 | 55.17 | 18374 | 15036 | 7888 | 7148 |
| S00474 | ASD | FC | 613378087 | 35377275 | 0.94 | 17.34 | 27.38 | 74.79 | 56.15 | 21018 | 17289 | 9574 | 7715 |
| S00475 | ASD | FC | 534658553 | 33790800 | 0.89 | 15.82 | 25.95 | 69.92 | 54.91 | 17710 | 14182 | 7462 | 6720 |
| S00480 | ASD | FC | 632774425 | 35211089 | 0.93 | 17.97 | 27.27 | 74.03 | 55.95 | 20441 | 17002 | 9211 | 7791 |
| S00512 | ASD | FC | 486591292 | 31308562 | 0.83 | 15.54 | 23.47 | 68.61 | 54.46 | 15149 | 12032 | 6362 | 5670 |
| S00514 | ASD | FC | 679952880 | 34863957 | 0.92 | 19.50 | 25.91 | 72.55 | 55.51 | 20015 | 16592 | 9237 | 7355 |
| S00518 | ASD | FC | 715701563 | 33792747 | 0.89 | 21.18 | 25.81 | 71.18 | 55.01 | 18740 | 15234 | 8445 | 6789 |
| S00560 | ASD | FC | 695324475 | 36369099 | 0.96 | 19.12 | 24.50 | 73.93 | 55.98 | 21052 | 18026 | 9946 | 8080 |
| S00563 | ASD | FC | 663458758 | 35801848 | 0.95 | 18.53 | 24.10 | 72.03 | 55.55 | 19764 | 16606 | 9091 | 7515 |
| S00564 | ASD | FC | 685598398 | 32770952 | 0.87 | 20.92 | 25.42 | 71.50 | 55.19 | 17282 | 14329 | 7860 | 6469 |
| S00569 | ASD | FC | 665237619 | 33101883 | 0.88 | 20.10 | 24.95 | 71.56 | 55.25 | 17200 | 14326 | 7637 | 6689 |
| S00607 | ASD | FC | 540051434 | 31924599 | 0.84 | 16.92 | 24.00 | 69.25 | 54.58 | 15874 | 12823 | 6887 | 5936 |
| S00609 | ASD | FC | 704793676 | 35322677 | 0.93 | 19.95 | 25.67 | 71.27 | 55.01 | 19972 | 16493 | 9153 | 7340 |
| S00613 | ASD | FC | 634337803 | 33477973 | 0.89 | 18.95 | 25.51 | 70.51 | 54.92 | 18206 | 14586 | 7993 | 6593 |
| S00655 | ASD | FC | 771094744 | 36481520 | 0.96 | 21.14 | 24.44 | 73.73 | 55.85 | 21433 | 18439 | 10119 | 8320 |
| S00658 | ASD | FC | 704800830 | 36007841 | 0.95 | 19.57 | 24.06 | 72.01 | 55.44 | 19770 | 16882 | 9489 | 7393 |
| S00701 | SCZ | FR | 1420423874 | 36911982 | 0.98 | 38.48 | 30.14 | 53.98 | 58.34 | 25398 | 22353 | 12644 | 9709 |
| S00714 | SCZ | FR | 1341566821 | 36777499 | 0.97 | 36.48 | 30.08 | 53.77 | 58.35 | 25180 | 22096 | 12613 | 9483 |
| S00724 | SCZ | FR | 645599908 | 35498250 | 0.94 | 18.19 | 28.37 | 50.69 | 58.07 | 22855 | 18307 | 9638 | 8669 |
| S00736 | ASD | FC | 495475072 | 35154901 | 0.93 | 14.09 | 23.44 | 65.73 | 54.11 | 17393 | 13977 | 7402 | 6575 |
| S00796 | SCZ | FR | 1481119845 | 36866069 | 0.98 | 40.18 | 30.10 | 53.92 | 58.31 | 25378 | 22330 | 12817 | 9513 |
| S00809 | SCZ | FR | 1467504368 | 37039306 | 0.98 | 39.62 | 30.36 | 54.09 | 58.32 | 25401 | 22560 | 12731 | 9829 |
| S00819 | SCZ | FR | 608028069 | 35666145 | 0.94 | 17.05 | 28.50 | 50.51 | 58.11 | 23094 | 18293 | 9524 | 8769 |
| S00831 | ASD | FC | 527645496 | 35768179 | 0.95 | 14.75 | 24.98 | 71.65 | 55.62 | 19066 | 15764 | 8151 | 7613 |
| S02092 | ASD | FC | 591890715 | 32224610 | 0.85 | 18.37 | 23.92 | 69.46 | 54.51 | 16740 | 13438 | 7475 | 5963 |
| S02187 | ASD | FC | 521565817 | 31731570 | 0.84 | 16.44 | 23.44 | 68.71 | 54.41 | 16037 | 12636 | 6793 | 5843 |
| S02352 | ASD | FC | 520320713 | 32324097 | 0.85 | 16.10 | 23.70 | 67.30 | 54.20 | 15917 | 12323 | 6598 | 5725 |
| S02447 | ASD | FC | 529948787 | 32342486 | 0.86 | 16.39 | 23.69 | 67.27 | 54.18 | 16370 | 12731 | 6801 | 5930 |
| S03170 | ASD | FC | 643883312 | 32842263 | 0.87 | 19.61 | 25.16 | 71.90 | 55.19 | 17532 | 14418 | 7971 | 6447 |
| S03171 | ASD | FC | 552204935 | 32639146 | 0.86 | 16.92 | 24.06 | 71.11 | 55.13 | 17161 | 13550 | 7271 | 6279 |
| S03204 | ASD | FC | 591204586 | 35644715 | 0.94 | 16.59 | 25.43 | 72.68 | 55.81 | 19501 | 16292 | 8938 | 7354 |
| S03265 | ASD | FC | 614973266 | 32941997 | 0.87 | 18.67 | 25.27 | 71.62 | 55.16 | 17460 | 14177 | 7732 | 6445 |
| S03299 | ASD | FC | 597872448 | 35595904 | 0.94 | 16.80 | 24.89 | 72.40 | 55.74 | 19967 | 16456 | 8918 | 7538 |
| S05706 | ASD | FC | 401394135 | 35126412 | 0.93 | 11.43 | 23.94 | 70.27 | 55.67 | 18538 | 14211 | 7207 | 7004 |
| S05712 | ASD | FC | 611714294 | 32735025 | 0.87 | 18.69 | 24.10 | 68.28 | 54.21 | 17098 | 13442 | 7412 | 6030 |
| S05801 | ASD | FC | 593401935 | 35685644 | 0.94 | 16.63 | 24.99 | 71.44 | 55.44 | 19933 | 16129 | 8540 | 7589 |
| S05804 | ASD | FC | 679756231 | 34216978 | 0.91 | 19.87 | 24.46 | 71.52 | 55.34 | 18013 | 14925 | 7965 | 6960 |
| S05807 | ASD | FC | 583203128 | 32340152 | 0.86 | 18.03 | 23.65 | 67.24 | 54.05 | 16376 | 12762 | 6979 | 5783 |
| S10812 | SCZ | FR | 666020409 | 35763013 | 0.95 | 18.62 | 28.19 | 50.83 | 58.05 | 23264 | 18784 | 9913 | 8871 |
| S10813 | SCZ | FR | 653938111 | 35943583 | 0.95 | 18.19 | 28.45 | 51.04 | 58.16 | 23980 | 19267 | 10157 | 9110 |
| S10815 | SCZ | FR | 753658592 | 35776136 | 0.95 | 21.07 | 28.47 | 51.45 | 58.13 | 23348 | 19354 | 10378 | 8976 |
| S10816 | SCZ | FR | 724677707 | 35665134 | 0.94 | 20.32 | 28.14 | 51.18 | 58.08 | 23308 | 18931 | 10860 | 8071 |
| S10818 | SCZ | FR | 834512767 | 35973418 | 0.95 | 23.20 | 28.62 | 51.81 | 58.12 | 23719 | 19908 | 10747 | 9161 |
| S10819 | SCZ | FR | 790482037 | 36104426 | 0.95 | 21.89 | 28.42 | 51.77 | 58.11 | 24284 | 20010 | 10873 | 9137 |
| S10821 | SCZ | FR | 1278934276 | 36824566 | 0.97 | 34.73 | 29.78 | 53.55 | 58.25 | 25059 | 22061 | 12623 | 9438 |
| S10822 | SCZ | FR | 1405108496 | 36705941 | 0.97 | 38.28 | 29.80 | 53.81 | 58.35 | 25540 | 22450 | 12899 | 9551 |
| S10824 | SCZ | FR | 1402537294 | 36869972 | 0.98 | 38.04 | 29.85 | 53.81 | 58.30 | 25329 | 22231 | 12816 | 9415 |
| S10825 | SCZ | FR | 1386245321 | 36766910 | 0.97 | 37.70 | 29.74 | 53.84 | 58.34 | 25567 | 22263 | 13122 | 9141 |
| S11506 | SCZ | FR | 1463310057 | 37050968 | 0.98 | 39.49 | 32.69 | 54.10 | 58.29 | 24894 | 22177 | 12426 | 9751 |
| S11507 | SCZ | FR | 1553954216 | 37034775 | 0.98 | 41.96 | 33.24 | 54.20 | 58.24 | 24497 | 22122 | 12517 | 9605 |
| S11509 | SCZ | FR | 1245732699 | 36867945 | 0.98 | 33.79 | 32.17 | 53.51 | 58.25 | 24252 | 21486 | 11875 | 9611 |
| S11510 | SCZ | FR | 1153204599 | 36854616 | 0.97 | 31.29 | 32.21 | 53.47 | 58.31 | 24170 | 21101 | 11865 | 9236 |
| S11512 | SCZ | FR | 1371119103 | 36775772 | 0.97 | 37.28 | 29.22 | 53.65 | 58.31 | 26204 | 22308 | 12875 | 9433 |
| S11513 | SCZ | FR | 1454692647 | 36564994 | 0.97 | 39.78 | 29.10 | 53.69 | 58.33 | 26000 | 22193 | 12587 | 9606 |
| S11515 | SCZ | FR | 1347218963 | 36761169 | 0.97 | 36.65 | 29.16 | 53.54 | 58.29 | 26475 | 22414 | 12466 | 9948 |
| S11516 | SCZ | FR | 1382914789 | 36520789 | 0.97 | 37.87 | 29.29 | 53.55 | 58.33 | 25665 | 21807 | 12408 | 9399 |
| S11518 | SCZ | FR | 1379528405 | 36476226 | 0.96 | 37.82 | 31.91 | 53.42 | 58.29 | 24184 | 21034 | 11621 | 9413 |
| S11519 | SCZ | FR | 1445884478 | 36656991 | 0.97 | 39.44 | 32.43 | 53.89 | 58.37 | 24890 | 21802 | 12388 | 9414 |
| S11521 | SCZ | FR | 1668600161 | 37024830 | 0.98 | 45.07 | 32.75 | 54.37 | 58.33 | 25348 | 22591 | 13020 | 9571 |
| S11522 | SCZ | FR | 1631713309 | 36511949 | 0.97 | 44.69 | 32.56 | 53.91 | 58.30 | 23961 | 21142 | 11913 | 9229 |
| S11524 | SCZ | FR | 1692981354 | 36829100 | 0.97 | 45.97 | 32.59 | 54.14 | 58.28 | 24806 | 22012 | 12440 | 9572 |
| S11525 | SCZ | FR | 1506592912 | 36936241 | 0.98 | 40.79 | 32.74 | 54.12 | 58.33 | 25154 | 22252 | 12650 | 9602 |
| S12240 | ASD | FC | 611418410 | 32924092 | 0.87 | 18.57 | 23.81 | 69.62 | 54.56 | 17437 | 13941 | 7801 | 6140 |
| S12252 | ASD | FC | 503163050 | 32879149 | 0.87 | 15.30 | 23.01 | 68.54 | 54.44 | 17152 | 13146 | 7182 | 5964 |
| S12253 | ASD | FC | 588534675 | 32888213 | 0.87 | 17.90 | 23.88 | 69.69 | 54.64 | 17661 | 14008 | 7989 | 6019 |
| S12271 | ASD | FC | 473153914 | 33004070 | 0.87 | 14.34 | 23.11 | 68.95 | 54.69 | 16720 | 12949 | 6875 | 6074 |
| S12272 | ASD | FC | 494986381 | 32189074 | 0.85 | 15.38 | 23.46 | 69.00 | 54.58 | 16840 | 12871 | 6897 | 5974 |
| S12584 | ASD | FC | 551001263 | 31807033 | 0.84 | 17.32 | 23.32 | 68.61 | 54.25 | 16238 | 12839 | 7072 | 5767 |
| S12586 | ASD | FC | 556132432 | 32044964 | 0.85 | 17.35 | 23.43 | 68.80 | 54.34 | 16579 | 12954 | 7109 | 5845 |

^*^ ALL, acute lymphoblastic leukemia; ASD, autistic spectrum disorder; PID, primary immunodeficiencies; SCZ, schizophrenia).

^†^ FC, French-Canadian; FR, French.
