# Supplementary material for: Whole-Exome Sequencing Reveals a Rapid Change in the Frequency of Rare Functional Variants in a Founding Population of Humans
Source: PLoS Genet. 2013 Sep 26;9(9):e1003815. doi: 10.1371/journal.pgen.1003815 (PMC3784517; doi:10.1371/journal.pgen.1003815)
Supplement: Table S6 — Results from forward simulations modeling the demographic histories of the French and French Canadian (FC) populations. (DOCX) [file pgen.1003815.s016.docx]

| **Bottleneck Size in FC** | **Increase in proportion of variants with MAF<5% in FC population** | **Largest increase in rare variants in French Canadians across 100 replicates** | **Average number of additional deleterious variants per replicate in FC population (per MB)** |
| --- | --- | --- | --- |
| 50% | 0.047% | 3.525% | 4.71 |
| 75% | 0.740% | 5.742% | 5.86 |
| 100% | 1.090% | 5.231% | 8.32 |
